# Physical Training Programs for Tactical Populations: Brief Systematic Review

**DOI:** 10.3390/healthcare11070967

**Published:** 2023-03-28

**Authors:** André Rasteiro, Vanessa Santos, Luís Miguel Massuça

**Affiliations:** 1 Higher Institute of Police Sciences and Internal Security, 1300-663 Lisbon, Portugal; andrerasteiro10@gmail.com; 2 Exercise and Health Laboratory, CIPER, Faculdade de Motricidade Humana, Universidade de Lisboa, 1495-751 Cruz Quebrada, Portugal; vstsantos@gmail.com; 3 KinesioLab, Research Unit in Human Movement Analysis, Instituto Piaget, 2805-059 Almada, Portugal; 4 ICPOL Research Center, Higher Institute of Police Sciences and Internal Security, 1300-663 Lisbon, Portugal; 5 Research Center for Sport, Physical Education, Exercise and Health, CIDEFES, Universidade Lusófona, 1749-024 Lisbon, Portugal; 6 CIFI2D, Faculty of Sport, Universidade do Porto, 4200-450 Porto, Portugal

**Keywords:** physical training programs, fitness assessments, health, law enforcement, muscular strength, tactical athletes

## Abstract

This review aims (i) to identify and analyze the physical training programs used for tactical personnel (TP) and (ii) to understand the effects of physical training programs on the health and fitness, and occupational performance of tactical personnel. A literature search used the keywords ‘Physical Training Program’, ‘Police’, ‘Law Enforcement’, and ‘Firefighter’. A total of 23 studies out of 11.508 analyzed were included. All studies showed acceptable methodological quality in assessing physical fitness (PF), and training programs’ effect sizes (Cohen’s *d*) on PF attributes were calculated. The results showed that physical training programs (duration > four weeks) can improve (medium-to-large effects) (i) measures of physical fitness and (ii) performance in simulations of occupationally specific tasks. This review provides summary information (i) to help select (or adjust) physical training programs for TP and (ii) to clarify the effect of different occupational-specific training interventions on fitness measures and health-related parameters for TP.

## 1. Introduction

Tactical populations (e.g., police officers, firefighters, and military) have their specific tasks, which are complex, varied in nature, unpredictable, and highly demanding from a physical fitness point of view [1].

This personnel executes, in the performance of their mission, a wide variety of actions, many of which are physical, where they may be required to: stop suspects, run, climb up/downstairs, pull, push, overcome obstacles, chase suspects, and use weapons from a vast panoply of options [2]. To perform these activities, tactical personnel require endurance, strength, speed, agility, and flexibility to undertake their profession [3].

To respond to this large number of actions and perform their mission efficiently, the tactical population (TP) must have a physical fitness (PF) that is up to the enormous challenges of the demanding professions. In addition, it is also of great importance that TP is in good PF condition. Otherwise, they can endanger the safety of the community or even their own safety [4].

There is considerable scientific evidence that the PF of this TP is below the general population and health recommendations [5,6,7]. It has been extensively studied and shown that physical components such as cardiorespiratory fitness, muscular strength, and others are closely related to health parameters and improved quality of life and, consequently, enhanced job skills [8,9,10]. In accordance, a decline in exercise practice has implications for the health of TP, which ultimately impacts the organizations themselves (lower productivity levels [11]), given they are one of their greatest assets.

Nevertheless, there is only one study on physical activity and the application of specific training programs in TP in Portugal. Therefore, this review aims (i) to identify and analyze the most used PF programs for TP and (ii) to understand their impact on the development of PF attributes associated with performing the function.

## 2. Materials and Methods

### 2.1. Experimental Approach to the Problem

The present work was conducted to identify the PF programs most used in scientific research with PT and to determine their impact on their physical abilities in performing their functions. The guidelines of the Preferred Reporting Items for Systematic Reviews and Meta-Analyses (PRISMA) model [12] were followed. The present study is exempt from ethical approval because the data came from previously conducted studies for which the authors of each study had obtained approvals.

### 2.2. Procedures

#### 2.2.1. Search Strategy

The author identified relevant original works for the literature search for this original work. To do this, literature databases were systematically searched using specific keywords pertinent to the topic, including PubMed (https://pubmed.ncbi.nlm.nih.gov/?term=Physical+Training+Program+AND+Police+OR+Law+Enforcement+or+Firefighter&filter=years.2012-2023&size=100, accessed on 7 March 2023) and SPORTDiscus|EBSCO (https://search.ebscohost.com/login.aspx?direct=true&db=s3h&bquery=Physical+Training+Program+AND+police+officers+OR+law+enforcement+OR+military+OR+firefighters&cli0=FT&clv0=Y&cli1=DT1&clv1=201201-202212&type=1&searchMode=Standard&site=ehost-live&scope=site”: EBSCOhost Research Databases) (accessed on 14 March 2023).

Databases were selected because they were high-quality, peer-reviewed articles that represented journals relevant to the topic of the study. We used specific terms and filters for the databases searched, which are summarized in Table 1.

**Figure 1:**
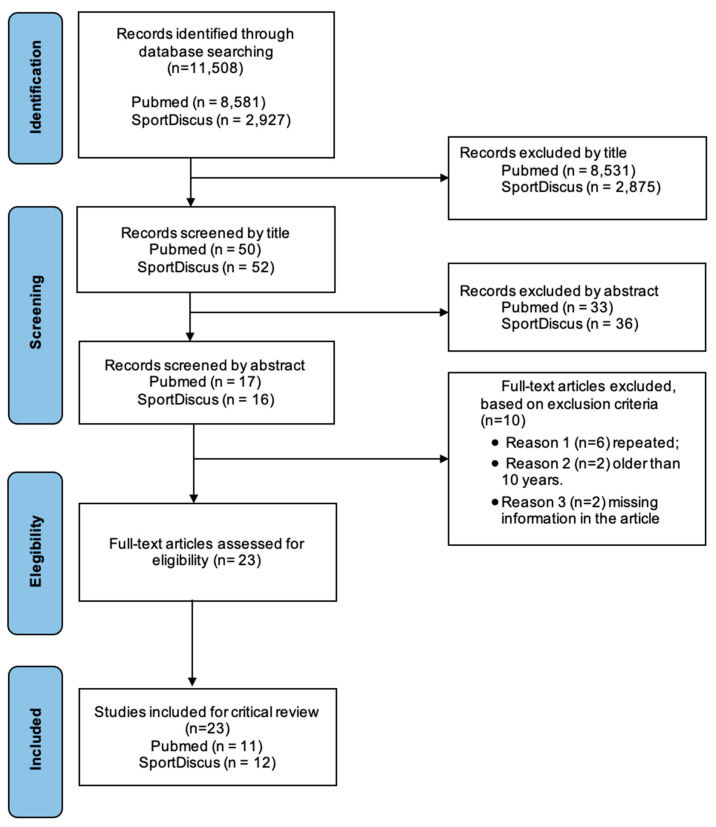
PRISMA diagram detailing the search process.

Eligibility criteria were defined and applied to each database to refine the search results. The defined inclusion criteria were individuals from police, fire, or other law enforcement agencies who have participated in a training program. The specified exclusion criteria were: (i) studies older than ten years; (ii) studies examining only body composition; and (iii) instrument development and validity studies. Duplicate studies were removed after all studies were collected. The screening and selection process is described in a PRISMA flow diagram (Figure 1) [12].

#### 2.2.2. Critical Appraisal

To assess the methodological quality of the studies, we used the NHLBI guidelines, which consist of a checklist of 14 questions. Each question can be answered “Yes”, “No”, “Not applicable”, “Not reported”, or “Cannot be determined”. Two authors also guaranteed methodological quality to avoid bias. Table 2 shows the quality of all studies in this review.

#### 2.2.3. Data Extraction

Afterwards, the articles were critically analysed, and the following information was extracted: authors and year of publication; study population; measurements (PF tests); physical training program; main results/general conclusions. All information is presented in Table 3. In continuation, the mean and standard deviations (SDs) for fitness test results (pre- and post-intervention) in each selected study were used to calculate the effect size (Cohen’s *d*) and effect size correlation (*r*) of the physical training programs on fitness measures (note that *d* and *r* are positive if the mean difference is in the predicted direction).

## 3. Results

### 3.1. Search Results

A total of 11,508 studies were identified. After being screened by titles, abstracts, and complete text analyses, 23 studies were considered (Table 3). We summarized the screening and selection process in the PRISMA flow diagram (Figure 1) and the literature search results [12].

The reviewed studies referred to TP/PO [13,28,30,32], firefighters [15,18,20,22,26,31,34], military [23,33], and cadets/recruits (police [3,16,19,21,25], firefighters [24,27,29] and military [14,17]).

Of the 23 studies, fourteen were realized in the USA [3,13,15,16,18,22,24,25,26,27,28,29,30,33], two from UAE [19,32], and one from South Africa [14], Brazil [17], Iran [20], Russia [21], Denmark [23], Portugal [31], and China [34].

Eight studies examined male and female participants [3,13,14,16,23,25,26,33], while twelve included only male participants [15,17,18,19,21,22,27,28,29,31,32,34]. Three studies did not report the gender of the participants [20,24,30].

### 3.2. Physical Fitness Measures

Morphological attributes (e.g., stature, body mass, body mass index—BMI, waist circumferences, hip circumferences, waist-to-height ratio, skinfolds, fat mass—%FM, or lean body mass) were assessed in eleven studies [16,17,19,22,24,27,29,30,31,32,33].

The most-used fitness components assessed were muscular strength (maximal strength, endurance, and power), aerobic capacity, anaerobic capacity (e.g., speed), agility, flexibility, and some specific professional tests, i.e.: (i) maximal muscular strength was measured in almost all studies in different forms, including bench press [3,16,29,33,34], leg press [33], squat [22,34], hex-bar deadlift [28], handgrip strength [3,15,21,25,27,30], and lower-back and leg strength [21,25,27]; (ii) muscular endurance was most measured by push-ups [3,14,16,17,19,21,22,23,24,25,26,29,30,32], sit-ups [3,14,16,17,19,23,25,26,29,30,32], pull-ups [22,24,27], and plank time [22,30]; (iii) muscular power was measured using vertical jump [3,16,25,27,29,34] and seated medicine-ball throw [34] tests; (iv) aerobic capacity measures were performed, a including 2.4-km run (1.5-mile run) [14,16,19,22,24,29,32], 20-m shuttle run [23,25,27,34] and 12-min run/Cooper [17,23,31] tests; (v) anaerobic capacity was measured using Wingate anaerobic [3] or sprint [3,16,28] tests; (vi) agility was tested with a T-test [3,32] and shuttle run [14,21]; (vii) flexibility was measured using the sit-and-reach test [3,15,26,29,30]; and (viii) specific tests were also measured in some studies [13,15,20,29,34], including victim drag/rescue [13,15,29], climbing rope [34], and others [18,20].

### 3.3. Physical Training Programs

**Figure 2:**
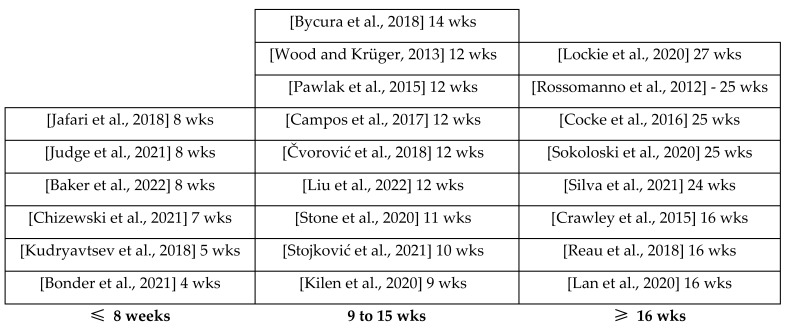
Physical training programs duration (in weeks; wks) [3,13,14,15,16,17,18,19,20,21,22,23,24,25,26,27,28,29,30,31,32,34].

The physical training programs applied in the studies ranged from four [28] to twenty-seven [25] weeks. Of the studies, three had a 25-week duration [13,16,26], five had a 12-week duration [14,15,17,19,34], three had 16-week [3,22,24] and 8-week [20,30,33] durations, and others had one article with five [21], seven [29], nine [23], ten [32], eleven [27], fourteen [18], and twenty-four [31] weeks duration. Figure 2 schematizes the time of the physical training programs. Additionally, as we can understand, most studies use physical training programs for 12- and 25-week durations.

The most-used PF programs were cardio training [3,13,14,15,16,17,18,19,22,23,24,25,26,27,29,31,32,33], weight training [3,14,15,16,17,19,20,21,22,24,27,30,31,32,34], calisthenics training (involving bodyweight exercises such as push-ups, pull-ups, and others) [3,13,15,16,19,23,24,25,26,29,30,32,33,34], and circuit training [3,13,15,17,19,22,23,25,26,29,30,31,32,33]. Three other studies were high-intensity functional training [16,29,33], and one applied the test repeatedly (20-m run and hex-bar deadlift) [28] (Table 4).

The results of this study have important implications for selecting the most-used physical training plans to improve exercise regimens for TP. We found that for the muscle endurance tests, such as the 60-s sit-ups and push-ups, training programs between 7 and 25 weeks showed large effect sizes (Cohen’s *d* between 0.99 and 5.65) [14,16,17,19,26,29,30,32], while the other tests showed small effects (Cohen’s *d* between 0.24 and 0.45) [3,23,28,29]. All abdominal muscle tests showed medium and small effects (Cohen’s *d* between 0.40 and 0.61) after 9 weeks of training [23], back extension showed medium and large effects (Cohen’s *d* between 0.77 and 1.03) after 9 weeks of exercise [23], while lunges test also showed small-to-medium effects (Cohen’s *d* between 0.45 and 0.78) after 9 weeks of training [23]. Regarding muscular strength, the 1 RM bench press test showed large effects at the 12 week intervention point (Cohen’s *d* ranging from 0.97 to 1.96) [34] and medium effects at the 25 week intervention point (Cohen’s *d* of 0.56) [16]. There were small effects at the 7 week intervention point (Cohen’s *d* ranging from 0.38 to 0.45) [34]; the 1 RM back squat, on the other hand, showed large effects (Cohen’s *d* between 0.82 and 1.25) at the 12 week intervention point [34]. Muscular power, countermovement jump with arm swing, and seated medicine ball throw showed large effects at the 12 week intervention point (Cohen’s *d* between 1.03 and 2.17) [34]; the vertical jump showed a medium effect at the 25 week intervention point in the RT group (Cohen’s *d*, 0.76) [16]; all other tests showed small and trivial effect values (Cohen’s *d* between 0.03 and 0.47) [16,29,34]. Flexibility at the 7 week intervention point showed a small effect (Cohen’s *d*, 0.31) [29] and a large effect at the 25 week intervention point (Cohen’s *d*, 0.93) [26]. Agility also showed small effects at the shorter intervention point of 10 weeks (Cohen’s *d*, 0.42) [3] and large effects at the intervention point of 16 weeks (Cohen’s *d*, 1.41) [32]. When we analyzed aerobic capacity variables, we found large and medium effects for most interventions between 7 and 25 weeks (Cohen’s *d* between 0.54 and 65.76) [16,19,23,29,32]. The anaerobic tests showed results with trivial effects in the sprint test at the 4 week intervention point (Cohen’s *d*, 0.18) [28], medium effects at the intervention point of 16 weeks (Cohen’s *d*, 0.51) [3], and small effects in the Wingate test (Cohen’s *d*, 0.42) [3]. Table 5 shows all results.

## 4. Discussion

The aims of this review were (i) to identify and analyze the most-used PF programs for TP and (ii) to understand their impact on the development of physical abilities associated with the performance of the function.

All studies showed acceptable methodological quality in assessing PF and the physical training program.

The tests assessing motor skills that greatly impacted task performance in the studies analyzed in this review varied widely. Of note were the tests assessing strength, which were present in 17 of the 23 studies analyzed [3,14,15,16,17,19,21,22,23,24,25,26,27,29,30,32,34], and aerobic capacity, which was present in 10 of 23 studies [14,16,19,22,23,24,25,27,29,32]. This was followed by the assessment of flexibility [3,15,26,29,30], speed [3,16,28,34], and agility [3,32]. The most applied assessments were: (i) handgrip test [3,15,21,25,27,30] and bench press [3,16,29,33,34] for muscle strength; (ii) push-ups [3,14,16,17,19,21,22,23,24,25,26,27,28,29,30,32] and sit-ups [3,14,16,17,19,23,25,26,29,30,32] for muscular endurance; (iii) vertical jump [3,16,25,27,29,34] for muscle power; (iv) 2.4-km run (1.5-mile run) [14,16,19,22,24,29,32] and 20-m shuttle run [23,25,27,34] for aerobic capacity; and (v) sit-and-reach [3,15,26,29,30] for flexibility.

Male TP performed significantly better than females on all measures [3,13,16,23,25,26], except for flexibility [3], measured through the sit-and-reach test.

The training plans applied in the different studies were diverse. The studies included in this review showed that a physical training program positively influences TP. The most-used PF programs were calisthenics/bodyweight training [13,15,16,17,20,24,25,26,27,30,31,33,34,35], cardio training [3,13,14,15,16,17,18,19,22,23,24,25,26,27,29,31,32,33], circuit training [3,13,15,17,22,23,25,26,29,30,31,32,33], and weight training [3,14,15,16,17,19,20,21,22,24,27,30,31,32,34]. Some other studies were high-intensity functional training [29], and one applied the test repeatedly (20-m shuttle run and hex-bar deadlift) [28].

In almost all programs, we observe a combination of various types of exercises, with body weight or using external loads (weights) combined with cardiovascular training.

Overall, the studies included in this review have shown that a physical training program could significantly improve tactical populations’ PF.

In the studies reviewed, statistically, significant improvements were seen in almost all [3,13,15,16,19,23,24,26,29,30,32,34], except for one [28], perhaps because the program was too short (4 weeks).

Despite the diversity and different options of the physical training programs, all of them proved fruitful since, in all the studies, improvements were observed in the motor skills evaluated and the health measures themselves.

In the study by Bonder et al. [28], they did not observe significant improvements in the sprint, perhaps because too short a training program (only four weeks) was applied, which could indicate that training programs in these areas need to be longer in duration or performed more times per week to provoke improvements, as noted by Lahti et al. [35], in a study they conducted with soccer players on speed. These authors suggest that training of at least eight weeks, 1 to 2× per week, should be applied to observe improvements. This is consistent with our findings, where most studies with more minor interventions had smaller effect sizes on PF performance tests.

We could conclude from this review that studies with less than eight weeks may not be sufficient to show significant differences [28]. Still, studies with more than 16 weeks are extensive and show little changes compared to TP between 9 and 15 weeks [15,19,23,32,34]. Thus, we can conclude that TP adjusted between 9 and 15 weeks show significant differences in PF [3,13,16,24,25,26].

In strength work, whether through a weight or bodyweight training program, we can see that improvements have been observed in short periods. Even in the study by Chizewski et al. [29], improvements were observed in only seven weeks. These results are like those obtained by Munn et al. [36], who also eyed improvements in strength capacity in only six weeks.

In the study by Cocke et al. [16], the randomized training group significantly improved all parameters. In contrast, the periodized group observed significant improvements in only three outcome measures (push-ups, sit-ups, and 300-m sprint). Periodized training does not provide additional improvements. Nevertheless, this information needs to be carefully analyzed as it contrasts with the study by Knapik et al. [37] that observed improvements in both periodized and randomized training groups.

Rossomanno et al. [13] and Lan et al. [24], who observed in their study several improvements in the training program applied after the end of the training program, when they reapplied the battery of tests sometime later, observed regression in the results obtained, both in the trials and in terms of health measures. In this sense, to ensure that police officers are prepared to perform their duties on the job, it is recommended that police departments provide a regular, supervised, job-based exercise program throughout the year [13].

Physical activity must be part of the daily routine for TP so that they improve or at least maintain high levels of PF that are essential for mission performance. The program must be supported throughout their lives because more is needed for TP to have physical activity during the course and not any physical activity at work.

However, a limitation of this review was the small number of studies analyzed. Initially, the idea was to critically review studies in which the sample consisted of police officers. However, after determining that there were very few studies of this type, it was decided to include studies in which the sample included so-called TP (i.e., tactical athletes). In addition to police officers, studies involving firefighters and military personnel were included, and studies involving cadets/recruits and cadets who are not yet TP were also included. Another limitation of these studies was the different methodological characteristics of each study (other test batteries), the different duration and frequency of use of the training, and the studies with different sexes when the results are presented in standard averages; therefore, the results here are weakened. This promotes considerable variability in the results with the small number of studies.

The content of this review is essential because it informs those responsible for developing training programs for tactical populations, which tests are most applied, and which training programs show the best results.

We consider it essential to develop a study like those analyzed [application of a training program to tactical populations] in Portugal to understand if the applications are transversal or if adaptations are necessary for the Portuguese context.

## 5. Conclusions

All studies included in this critical review have been evaluated as fair-to-sound quality, proving that training programs of varied frequency and exercise type can help improve required fitness testing results and optimize job performance.

To be effective, physical training programs should last at least eight weeks and have a weekly frequency of at least three times. Programs that combined strength training with cardiovascular training were shown to be more effective in creating positive changes in outcome measures and included exercises such as push-ups, running, bench press, front and back squats, burpees, lunges, sprints, and work-specific simulations (e.g., loaded run and dummy drag).

Because of their physically demanding work, TP needs specific training programs for their activity, which must remain throughout their career.

After a survey of studies conducted in this scope, only one investigation was observed in Portugal with a training program applied to TP. Therefore, conducting more studies to provide TP with adequate exercise for their functions is necessary. It is also essential to conduct a study with the long-term fitness and health outcomes of a randomized vs. periodized approach to clarify if traditional programs provide (or not) additional benefits over periodized exercise programs.

## Figures and Tables

**Table 1:** Databases and relevant search terms.

Databases	Search Terms	Filters (Sort By)	Results
PubMed	“Physical Training Program” AND “Police” OR “Law Enforcement” OR “Firefighter”	Best Match	8581
SPORTDiscus|EBSCO	“Physical Training Program” AND “Police” OR “Law enforcement” OR “military” OR “firefighters”	Relevance	2927

**Table 2:** NHLBI quality control tool items and study scores (n = 23).

Study	Item 1	Item 2	Item 3	Item 4	Item 5	Item 6	Item 7	Item 8	Item 9	Item 10	Item 11	Item 12	Item 13	Item 14	Score
Rossomanno et al., 2012 [13]	Yes	Yes	NA	Yes	No	Yes	Yes	NA	NA	Yes	Yes	Yes	Yes	No	9
Wood and Krüger, 2013 [14]	Yes	Yes	NA	Yes	No	Yes	Yes	No	Yes	Yes	Yes	Yes	Yes	No	10
Crawley et al., 2015 [3]	Yes	Yes	NA	Yes	No	Yes	Yes	No	Yes	Yes	Yes	Yes	Yes	No	10
Pawlak et al., 2015 [15]	Yes	Yes	NA	Yes	Yes	Yes	Yes	No	Yes	No	Yes	Yes	Yes	No	10
Cocke et al., 2016 [16]	Yes	Yes	NA	Yes	No	Yes	Yes	No	Yes	No	Yes	Yes	Yes	No	9
Campos et al., 2017 [17]	Yes	Yes	NA	Yes	No	Yes	Yes	No	Yes	No	Yes	Yes	Yes	No	9
Bycura et al., 2018 [18]	Yes	Yes	NA	Yes	No	Yes	Yes	No	Yes	No	Yes	Yes	Yes	No	9
Čvorović et al., 2018 [19]	Yes	Yes	NA	Yes	Yes	Yes	Yes	No	Yes	Yes	Yes	Yes	Yes	No	11
Jafari et al., 2018 [20]	Yes	Yes	NA	Yes	No	Yes	Yes	No	Yes	No	Yes	Yes	Yes	No	9
Kudryavtsev et al., 2018 [21]	Yes	Yes	NA	Yes	No	Yes	No	No	Yes	No	Yes	Yes	Yes	No	8
Reau et al., 2018 [22]	Yes	Yes	NA	Yes	No	Yes	Yes	No	Yes	No	Yes	Yes	Yes	No	9
Kilen et al., 2020 [23]	Yes	Yes	NA	Yes	No	Yes	Yes	No	Yes	No	Yes	Yes	Yes	No	9
Lan et al., 2020 [24]	Yes	Yes	NA	Yes	No	Yes	Yes	No	Yes	Yes	Yes	Yes	Yes	No	10
Lockie et al., 2020 [25]	Yes	Yes	NA	Yes	No	Yes	Yes	No	Yes	Yes	Yes	Yes	Yes	No	10
Sokoloski et al., 2020 [26]	Yes	Yes	NA	Yes	No	Yes	Yes	No	Yes	No	Yes	Yes	Yes	No	9
Stone et al., 2020 [27]	Yes	Yes	NA	Yes	No	Yes	Yes	No	Yes	No	Yes	Yes	Yes	No	9
Bonder et al., 2021 [28]	Yes	Yes	NA	Yes	No	Yes	No	No	Yes	No	Yes	Yes	Yes	No	8
Chizewski et al., 2021 [29]	Yes	Yes	NA	Yes	No	Yes	No	No	Yes	No	Yes	Yes	Yes	No	8
Judge et al., 2021 [30]	Yes	Yes	NA	No	No	Yes	Yes	No	Yes	No	Yes	Yes	Yes	No	8
Silva et al., 2021 [31]	Yes	Yes	NA	Yes	No	Yes	Yes	No	Yes	No	Yes	Yes	Yes	No	9
Stojković et al., 2021 [32]	Yes	Yes	NA	Yes	No	Yes	Yes	No	Yes	No	Yes	Yes	Yes	No	9
Baker et al., 2022 [33]	Yes	Yes	NA	Yes	No	Yes	Yes	No	Yes	Yes	Yes	Yes	Yes	No	10
Liu et al., 2022 [34]	Yes	Yes	NA	Yes	Yes	Yes	Yes	No	Yes	No	Yes	Yes	Yes	No	10

Note: key questions and NHBLI quality control tool items are available from: https://www.nhlbi.nih.gov/health-topics/study-quality-assessment-tools (accessed on 14 March 2023). Key: NA, “Not applicable”.

**Table 3:** The data extraction table, including physical fitness tests and training programs, with key findings.

Reference	Population	Measures/Physical Fitness Tests	Physical Training Program	Main Results/General Conclusions
Rossomanno et al., 2012 [13]	PO Young overweight USA n = 165 ♂, n = 131 ♀, n = 34	PAT: Running Jump over a 1-foot hurdle Jump over a 2-foot hurdle 4-foot long jump Walk down a 6-inch wide, 8-foot long beam Fall down, touch chest to floor, stand up Drop to your back, touch your shoulder blades to the floor, and stand up Climb over a wall 4 feet high Climb up and down 6 flights of stairs 75-lb push, walk in a half circle, 75-lb pull, Walk in a half circle 150-lb dummy 50-ft pull Sprint 50 yd Dry fire a gun 5x with each hand	25 wks. Aerobic training (brisk walking): Increase from 3 d/wk, 20 min/session at 60% of HRR to 5 d/wk, 30 min/session at 75% of HRR after 3 months. Calisthenics exercises: 3 d/wk (2 sets of 5 reps with own BW) to 5 d/wk (3 sets of 15 reps with own BW) after 3 months.	A supervised exercise program effectively improved body composition and cardiovascular and muscular fitness in PO. The exercise program was effective for both sexes.
Wood and Krüger, 2013 [14]	Military recruits South Africa NCPG ♂, n = 73 ♀, n = 115 CPG ♂, n = 100 ♀, n = 85	2.4-km run 4-km walk Sit-ups Push-ups Shuttle run test (10 × 22-m)	12 wks. Both groups, except for a different physical training program, followed the same BMT. Activities included drill, regimental aspects, compliments and saluting, general military aspects, musketry, shooting, signal training, mine awareness, map reading, buddy aid, field craft, water orientation, parade rehearsal, and physical training.	New cyclic-progressive PT program elicited more change in fitness parameters as measured via the Standardised Fitness Test than the traditional PT program, although it only yielded superior performance at final measurement in the men’s push-up.
Crawley et al., 2015 [3]	Police Cadets USA n = 68 ♂, n = 61 ♀, n = 7	Sprint (40-yds) Push-ups (60 s) Sit-ups (60 s) Handgrip 1 RM bench press Vertical jump Shuttle run (1/2-mile) *t*-Test Sit-and-reach Arm crank (PPO) Wingate (PPO)	16 wks (3 d/wk). Monday: outside group run (2 miles); Calisthenics routine (60 s, 1–3 sets—half squat; push-ups; pull-ups; chin-ups; sit-ups/crunches; back extensions; heel raises). Wednesday: plyometric exercises (1 set of 10 reps with 3 min of slow walking between each exercise—box jumps; split squat jumps; vertical power jump with both legs; single, double, and alt leg hops; clap push-up); weight training (2–3 sets of 8–12 reps, R: 1 min—leg press; leg extensions; leg curls lying down; lat pulldown; seated rowing; bench press; shoulder press; triceps press and biceps curls; calf raises; abdominal curls and back extensions). Friday: obstacle course (push-ups × 60 s; dummy drag; 95-pound bag carry; half-mile shuttle run for time); Track sprints (8 × 220 m in ≤42 s, R: 2 min between each rep).	Evidence of improvement in physical fitness in the first 8 wks. None of the variables showed significant improvement in the second 8 wks.
Pawlak et al., 2015 [15]	Firefighters Professional USA ♂, n = 20 SEG, n = 11 CG, n = 9	Handgrip Sit-and-reach SFGT: Tower climb Hose hoist Forcible entry simulation Ladder raise Hose advance Victim rescue	12 wks. Workout: general warm-up, dynamic stretching, circuit training, strength and endurance exercises, cardiovascular training, and static flexibility training. 3 mesocycles: 1st (wks 1–4)—30 s of work and 30 s of rest; 2nd (wks 5–8)—30 s of work and 15 s of rest; 3rd (wks 9–12)—30 s of work and 0 s of rest.	The SEG improved the completion rate on a standardized SFGT from 82 to 100% after the intervention, whereas the CG declined from 78 to 56%. The linear periodized training program improved firefighter physical ability in 1.5%. Those completing probationary follow-up (45/92 recruits) showed that most health/fitness improvements declined after graduation.
Cocke et al., 2016 [16]	Police Cadets USA n = 90 ♂, n = 70 ♀, n = 20 Groups: RaT1, n = 18 RaT2, n = 14 RaT3, n = 15 RaT4, n = 18 PT, n = 25	Body mass Fat mass Lean body mass Push-ups (60 s) Sit-ups (60 s) Bench press Vertical jump 2.4-km run 300-m sprint	25 wks, 5 days/wk. Total duration of each session: 60 min. RaT: includes strength and endurance exercises with a focus on improving fitness assessment performance. High repetitions of push-ups, sit-ups, pull-ups, and high-intensity metabolic conditioning style training. PT: phases designed to increase endurance, hypertrophy, strength, or power for overall health and physical conditioning rather than specifically for fitness testing. All fitness workouts: Warm-up: ~10 min, increasing intensity and stretching. Cooldown: ~10 min; emphasis on static stretching.	A program with a variety of training exercises showed better short-term improvement in fitness scores than a specifically structured training program focusing on individual performance areas. Long-term fitness and health outcomes are needed to prepare for a career as a PO, not just to pass initial fitness tests.
Campos et al., 2017 [17]	Air Force Recruits Brazil ♂, n = 130	Body mass Skinfolds thickness Circumferences Body fat Lean body mass Sit-ups (60 s) Push-ups Aerobic power test (12 min protocol)	12 wks, 32 sessions, 90 min/session. Distributed into cardiopulmonary and neuromuscular training sessions. Training period, sessions were used involving short, medium, and long runs (continuous and interval), stretching and localized exercises (e.g., push-ups, sit-ups, squat, single leg squat, basic plank, elbow plank, and jumping jacks).	Physical training carried out based on the Brazilian army manual causes alterations in morphological and physical fitness. 12 wks periodized physical training is a factor in chronic adaptations in body composition and physical fitness of the military.
Bycura et al., 2018 [18]	Firefighters USA ♂, n = 20 GSIP intervention arm, n = 12 CG (passive control arm), n = 8	Cosmed K4b2: 8 tasks repeated for 15 min.	14 wks, 3–5 days per week, 20–60 min in duration at 40–85% of heart rate. GSIP group: ACSM guidelines.	Compared to the CG, the GSIP intervention did not produce improvements in cardiovascular health. Subjects in both experimental conditions exhibited significant improvements in 2 of the 3 outcomes (i.e., *V*O2 and RER). A 14 wks period of time encouraged subjects to engage in a higher level of exercise overall in preparation to perform well.
Čvorović et al., 2018 [19]	Police Cadets Adu Dhabi UAE ♂, n = 325	Body mass Body composition Waist circumference Waist-to-height ratio Push-ups (60 s) Sit-ups (60 s) 2.4-km run	12 wks, 2 mesocycles: 6 + 6 wks. 1st mesocycle: physical training consisted of circuit training with BW exercises: 2nd mesocycle: increase in training volume and intensity through supersets and low to moderate load plyometric exercises.	A well-structured exercise program can be a means to continuously increase fitness. Training may not be optimal for participants with already high skills and abilities.
Jafari et al., 2018 [20]	Firefighters Iran n= 522 (does not mention the gender of participants) EG, n= 51 CG, n= 45	FMS NASM protocol	8 wks, 3 sessions of 1 hr/wk. CG: followed their own routine program, which consisted of endurance and resistive training. EG: training protocol based on NASM guidelines. Six stages: warming up, inhibiting, lengthening, activating, integrating, and cooling down training. The training was modified to extent every 2 wks.	43% of the participants scored lower than the critical FMS value of 14. The study shows they have insufficient functional fitness for their occupational activities in times of danger and that they have a higher potential of injury risk.
Kudryavtsev et al., 2018 [21]	Siberian Law Cadets Russia ♂, n = 28 Groups: Control, n= 14 Experimental, n=14	Dineika test Timed inspiratory capacity Romberg test LVC HR Harvard step-test Handgrip Lower-back and leg strength Turning up Shuttle run (10 × 10-m) Lifting Push-ups Half level position	5 wks, 90 min/session. All fitness workouts: warm-up (~20 min; active muscular activity); main part (~45–50 min); flexibility (~10 min). Program of classes: various exercises burdens with a barbell, weights, dumbbells.	Insufficient physical fitness of the young people for the future professional activities. Should have adaptation of the modern techniques of intensive functional training (CrossFit) in the process of physical training of the cadets and military students. The purposeful implementation of CrossFit-style exercises, which effectively improve strength and cardiorespiratory fitness, can significantly enhance the speed-strength, weightlifting, and functional abilities of future officers and PO within a relatively short timeframe of 4–5 wks.
Reau et al., 2018 [22]	Firefighters USA ♂, n = 148	Body mass KPI testing: Squats Push-ups Pull-ups Plank 2.4-km (1.5-mile) run	16 wks, 4/wk for 90 min. Program: incorporated a warm-up, endurance training, strength training, and a cool-down/recovery period. Three parts: Prepare, Sweat, and Recover Prepare: pillar activation, chain activation, and anatomical alignment exercises were used. Sweat: exercises enhance triple extension speed, lower body push-pull movements, upper body push-pull movements, and horizontal and vertical conditioning movements that stressed the aerobic and anaerobic energy systems. Recover: consisted of foam rolling and static stretching.	16-wk progressive training program reveals that overall indices of physical fitness improved in more than 89% of the population, depending on the specific fitness outcome. At 8 wks into the program while scores showed improvement and continued over the 16 wk period.
Kilen et al., 2020 [23]	Military Conscripts Denmark n = 290 ♂, n = 286 ♀, n = 4	Push-ups (120 s) Sit-ups (120 s) Lunges (120 s) Back extension 20-m shuttle run 12-min run	9 wks. MIC: 15 min-endurance training blocks and four 15-min strength training blocks. CLA: 60-min endurance or strength training blocks, matched for exercise type and intensity. Interventional training: two 60-min sessions as a standard military basic training fitness program with mixed exercises, i.e.,: ∼40% strength training (blocks: 3 sets of multijoin exercise × 5 reps); ∼60% running [(i) moderate pace running (wks 1–3), (ii) 60–120 s intervals with equal ratio of work to rest (wks 4–6), and (iii) 30 s intervals of high intensity with 3 min rest in between (wks 7–9)] or muscle endurance training [three rounds of five exercises (two lower extremity, one upper body, one upper body, and one flexibility; 5 × 30 s, 30 s rest between exercises)].	Frequent 15-min workouts were not superior to 60-min workouts for improving running performance and strength endurance. Increases in 12-min running capacity and shuttle run performance were similar between MIC and CLA. Muscular endurance training increased multi-joint exercise capacity by ∼3-fold in untrained women after 4 wks. Short, frequent exercise sessions appear to be a viable training strategy when time is limited.
Lan et al., 2020 [24]	Firefighter Recruits New England, USA n = 92 (does not mention the gender of participants)	BP BMI %FM Push-ups (60 s) Pull-ups (max) 2.4-km (1.5-mile) run	16 wks, 4 days/wk. Program: 8 to 10-min warm-up exercises; Intensive physical training (cardiorespiratory training or muscular strength and muscular endurance, interval runs/sprints); resistance training and core muscle strengthening; R: 10 to 15 min (cool-down and flexibility exercises).	Fire academy training has been shown to improve recruit body composition and some measures of physical fitness, and to promote healthy lifestyles. The probationary period negatively impacted recruits’ BMI, %FM, push-ups, physical activity scores, and TV screen time. Recruits’ BP increased throughout the study period.
Lockie et al., 2020 [25]	LEO Recruits USA n = 26 ♂, n = 23 ♀, n = 3	Push-ups (60 s) Sit-ups (60 s) Handgrip Vertical jump Lower-back and leg strength 20-m shuttle run	27 wks, 45 min session. Tuesday: power clean/front squat × 3, bent over rows × 5, push-ups × 7. Wednesday: burpee pullups for maximum rep. Thursday: sprints × 10, suicide sprints × 10, beep test. Friday: wall throws with ball, broad jump burpees, kettlebell swings, front squats (×21; ×15; ×9).	The strength and conditioning program improved most fitness parameters. Push-ups, sit-ups, MSR improved from pre- to post-test but not from mid- (14 wks) to post-test. Apart from handgrip, all tests improved from pre- to post-test. Lower body strength and power improved from mid- to post-test.
Sokoloski et al., 2020 [26]	Firefighters Professional USA n = 34 ♂, n = 32 ♀, n = 2	Push-ups (max) Sit-ups (60 s) Sit-and-reach	25 wks (6 months), 2 d/wk. Circuit training: wk1 (d1 and d2) dynamic warmup; wk2 (d1) gilbert squat 3 × 5, push-up 3 × 10, band pull apart; wk2 (d2) KB swing 3 × 5, banded row 3 × 10, farmers carry 3 × 20-yds; wk3 (d1) landmine deadlift 3 × 5, military press 3 × 8, plank 3 × 30 s; wk3 (d2) Jacob’s ladder 3 × 30 s, beep test; wk4 (d1) box jumps 2 × 5, trap bar deadlift 3 × 5, side plank 3 × 30 s; wk4 (d2) DB BP 3 × 5, DB row 3 × 8, good mornings 2 × 10; wk5 (d1) 10 × 15 s: banded KB swings, banded good mornings, farmers walks; wk5 (d2) 10 × 15 s: push-ups, med ball depth drop toss, maximal-effort plank; wk6 (d1) 8 × 15 s each: tire flips, sledgehammer alternating hits, farmers walk; wk6 (d2) 3 × 60 s: KB swing, reverse lunge and press, plank; wk7 (d1) plyometric push-up 3 × 10, trap bar deadlift 5 × 5, SA farmers walk 1 × 120 s; wk7 (d2) box jump 5 × 5, military press 5 × 5, DB row 6 × 10; wk8 (d1) 3 × 60 s: tire flips SA farmers walk, sledgehammer alternating hits, sled pull; wk8 (d2) 4 × 120 s: Jacob’s ladder, beep test; wk9 (d1) 3 × 120 s: banded KB swing, Jacob’s ladder; wk9 (d2) Military press 5 × 8, prone row 4 × 12, beep test; wk10 (d1) 3 × 120 s: DB step-up, side plank; wk10 (d2) 6 × 30 s: banded row, push-ups; wk11 (d1) DB incline press 5 × 5, AMRAP (≤10-min), BB deadlift × 5, inverse row × 5, push-ups × 5; wk11 (d2) trap bar deadlift 5 × 5, AMRAP (≤10-min), BO DB row × 5, DB military press × 5, med ball slam × 5; wk12 (d1) landmine deadlift 5 × 12, good mornings 4 × 8, beep test; wk12 (d2) landmine press 5 × 12, DB row 5 × 8, 3 × 400 m run; wk13 (d1) 6 × 30 s: tire flips sledgehammer alternating, hits, farmers walk; wk13 (d2) beep test × 2; wk14 (d1) AMRAP × 2 (≤5 min each), DB sumo squat × 8, DB bent over row × 8, trap bar deadlift × 8, farmers walk (20-yds); wk14 (d2) AMRAP × 2 (≤5 min each), push-ups x8, banded row × 10, band pull apart × 15, med ball slam × 8; wk15 (d1) 6 × 30 s each: DB sumo squat × 8, DB bent over row × 8, trap bar deadlift × 8, farmers walk (20-yds); wk15 (d2) 6 × 30 s: push-ups, banded row, band pull apart; wk16 (d1) 6 × 30 s: landmine deadlift, landmine press, sled drag, DB BP; wk16 (d2) 6 × 30 s: goblet squat, DB push press, banded row, battle ropes; wk17 (d1) 3 × 60 s: tire flips, SA farmers walk, sledgehammer alternating hits, sled pull; wk17 (d2) 4 × 120 s: Jacob’s ladder, beep test; wk18 (d1) 10 × 15 s: banded KB swing, banded good mornings, farmers walks; wk18 (d2) 10 × 15 s: push-ups, med ball depth drop toss, maximal-effort plank; wk19 (d1) 3 × 120 s: DB step-up, side plank; wk19 (d2) military press 5 × 8, prone row 4 × 12, beep test; wk20 (d1) 3 × 120 s: DB step-up, side plank; wk20 (d2) 6 × 30 s: banded row, push-ups; wk21 (d1) DB incline press 5 × 5, AMRAP (≤10-min), BB deadlift × 5, inverse row × 5, push-ups × 5; wk21 (d2) trap bar deadlift 5 × 5, AMRAP (≤10-min), BO DB row × 5, DB military press × 5, med ball slam × 5; wk22 (d1) landmine deadlift 5 × 12, good morning 4 × 8, beep test; wk22 (d2) landmine press 5 × 12, DB row 5 × 8, 3 × 400 m run; wk23 (d1) 6 × 30 s: tire flips, sledgehammer alternating hits, farmers walk; wk23 (d2) beep test × 2; wk24 (d1) 6 × 30 s: tire flip sled pull, alternating sledgehammer hits; wk24 (d2) 6 × 30 s: push-ups, banded row, band pull apart; wk25 (d1) 3 × 20 s: push-ups, banded row, goblet squat, med ball slams; wk25 (d2) beep test.	Exercise training appears to be a beneficial method for improving health-related physical fitness in professional firefighters.
Stone et al., 2020 [27]	Firefighter trainees USA ♂, n = 23	Stature Body mass (BW) BMI Pull-up Handgrip Lower-back and leg strength Vertical jump 20-m shuttle run	11 wks, three 12 h/day and one 4 h/day with 75 min of formal physical training on two of the days. Formal training: consisted of a dynamic warm-up (~10–12 min), agility training (~7–8 min), speed and power training (~3–4 min), hypertrophy/strength training (~30–35 min), trunk, mobility, and conditioning (~5–10 min), and a cooldown (~5 min). Additionally, performed an aerobic fitness session, interspersed with callisthenic exercises, as a group, once per week for approximately 60 min.	Significant improvements in both BW and BMI were observed. Improvements in upper-body strength and endurance as well as lower-body maximal and relative strength, and also on 20-m shuttle run. No significant changes were found for grip strength, VJ height, or lower-body power. VJ height has been shown to correlate to job task performance within firefighting populations. No changes in grip strength were observed over the 11-wk training period. The study shows that an 11-wk strength and conditioning program with minimal resistance training equipment, in addition to standard fire academy training, improves the physical fitness of firefighter trainees.
Bonder et al., 2021 [28]	LEO USA ♂, n = 7	20-m sprint HBD	4 wks (3 d/wk). Training: Standardized dynamic warm-up; 4-sets of 3 reps on the HBD; Four 20-m sprints (no longer than 15 min).	Maximal relative strength of the lower body was significantly improved by the occupation-specific training program. No improvements were demonstrated in the 20-m sprint.
Chizewski et al., 2021 [29]	Firefighters Recruits USA ♂, n = 89	Stature Body mass Push-ups (60 s) Sit-ups (60 s) Bench press Vertical jump 2.4-km (1.5-mile) run Sit-and-reach Kiser sled SCBA crawl Victim drag Hose advance Equipment carry Ladder raise Challenge total	7 wks (5 d/wk). Training: 10-min dynamic warm-up (jumping jacks, jump rope, and dynamic stretching); 40 min of HIFT (muscular strength and endurance, power, aerobic capacity, agility, and flexibility).	Several components of physical fitness were related to better and faster performance in simulated fire scene activities. Cardiovascular endurance and muscular endurance were the strongest predictors of rapid completion of such tasks.
Judge et al., 2021 [30]	PO & Ball State University students USA n = 38 (does not mention the gender of participants) Groups: Students, n = 22 PO, n = 16	Resting HR and BP Stature Body mass BMI Waist and hip circumferences 3-site skinfold %FM Push-ups (60 s) Sit-ups (60 s) Handgrip Sit-and-reach Plank hold YMCA step test	8 wks, 2 d/wk, 60 min/session. Day 1. legs: front squats (3 × 10, R: 3 min), leg press (3 × 10, R: 1–2 min), standing good morning (3 × 10, R: 1–2 min), step-ups (5 × 5 jumps, R: 1–2 min), lunges (3 × 10 each side, R: 1–2 min). Day 2. arms: BP (3 × 10, R: 1–2 min), lat pull down (3 × 10, R: 1–2 min), biceps curls (3 × 10, R: 1–2 min), triceps press (3 × 10, R: 1–2 min), push-ups (3 × 8(burnout first wk), R: 1–2-min). Day 3. participant training program—core/flexibility: knee hugs (3 × 8, R: 1–2 min), crunches (3 × 10, R: 1–2 min), jump rope (3 × 30 s, R: 3 min), Russian twist (3 × 8, R: 1–2 min), medicine ball slams (3 × 8, R: 3 min), plank (3 × 15 s, R: 1–2).	PO showed significant improvements in core and upper body muscular endurance. Resting HR, systolic and diastolic BP, BW, BMI, waist and hip circumferences, sum of skinfolds, and %FM improved at the end of the program (8 wks).
Silva et al., 2021 [31]	Firefighters Portugal ♂, n = 60 Groups: Training with PPE + SCBA (EG1) Training with regular equipment (EG2) Control group	Stature Body mass Cooper test	24 wks, 2 sessions/wk, with 4 phases each. Phase 1 (mesocycle adaptation) lasted 4 wks; Phase 2 (mesocycle gain 1) took 8 wks; Phase 3 (mesocycle gain 2) took 4 wks; Phase 4 (mesocycle improvement) lasted 8 wks. Training program included 12 functional fitness exercises: combined aerobic, BW, and weightlifting exercises designed to use the available equipment in a fire station (e.g., weight racks, benches) or on the fire ground (e.g., carrying equipment, dragging a dummy)	Implemented specific physical fitness program was relevant in the improvement of firefighters’ cardiorespiratory fitness independent of training modality. EG1 had greatest increase, observed as % of difference and effect size, when compared to EG2 and CG. A modern functional training, based on professional functions, enhances cardiorespiratory fitness. Regular functional training with PPE+SCAB must be encouraged to improve adequate physical fitness and *V*O_2max_, developing a healthy general physical condition and optimum fitness levels related to firefighting-specific tasks.
Stojković et al., 2021 [32]	PO UAE Overweight or obese ^A^ ♂, n = 46	Stature Body mass Push-ups (60 s) Sit-ups (60 s) 2.4-km run *t*-test	10 wks (5 d/wk; twice a day). Sunday: bodyweight and cardio training (am)—4 × 30 s, R: 3 min (outdoor running—2 km; push-ups + jumping jacks; sit-ups + mountain climbers; squat + burpees); flexibility and mobility exercises (pm)—30 s (lower and upper body stretches; trunk stretches; upper and lower body mobility exercises). Monday: strength circuit training (am)—3 × 12, R: 2 min (outdoor running—1 km; triceps press; shoulder press; squat—viper; biceps curl; lunges—weight); trunk stability and static stretching (pm)—3×, R: 3 min (leg lift; bicycle crunch; Russian twist; 15-cm hold; superman; plank). Tuesday: 2.4-/4-km trial running and dynamic stretching (am); Bodyweight training (pm)—4×, R: 2 min (squat thrusters; triceps dips; reverse lunges; glute bridge). Wednesday: bodyweight and cardio training (am)—3 × 1-min, R: 2 min (outdoor running—1 km; step ups; deck squats; back extensions; heel raises; wall ball; plank); agility training (pm) 8 min each station, R: 3 min (agility ladder lateral jumps; hurdles drills; lateral shuffles with cones; 10-m sprint). Thursday: bodyweight circuit training (am)—2–3 × 8–10 min, R: 3 min (outdoor running—2 km; 10 × push-ups; 20 × burpees; 30 × squat; 40 × sit-ups; 40 × sit-ups; 30 × squat; 20 × burpees; 10 × push-ups).	The training program has greatly improved anthropometric attributes and physical abilities (in a relatively short period of time).
Baker et al., 2022 [33]	Military (ROTC) USA n= 18 ♂, n = 14 ♀, n = 4 CG: n= 18	Fasted blood draw DXA pQCT scan 1 RM bench press 1 RM leg press Maximal aerobic capacity test	8 wks. The exercise routine consists of high-intensity interval, resistance, and aerobic training, and all 16 training sessions are designed to incorporate all 3 types of exercises. The circuit is completed twice and followed by a 3-min run covering 4.8 km.	Positive effects were found on bone after 8 weeks of ROTC training. In the ROTC group, sclerostin combined with measures of body composition and physical performance predicted 46 to 66% of estimated bone strength variance at the fracture-prone 38% tibia site, whereas PTH was less consistently predictive. Muscular strength increased from pre- to mid-intervention for both groups; however, these measures either plateaued or returned to baseline values by post-intervention. Was found positive body composition changes in both the ROTC and CG.
Liu et al., 2022 [34]	Firefighters Professional China ♂, n = 30 Groups: CT, n = 15 CG, n = 15	100-m load-bearing run 60-m shoulder ladder run 4th-floor climbing rope Vertical jump (Abalakov) Seated medicine ball throw 1 RM bench press 1 RM back squat 20-m shuttle run	12 wks (3 × 4 wks) Stages: I, 75% 1 RM; II, 80% 1 RM; III, 85% 1 RM. CT Program (3 series × reps 4~6 + 10~12): 1st and 2nd wks Monday: squat + SJ + barbell bench press + high-five push-ups. Thursday: deadlift + high pull+ loaded pull-ups + elastic band pull-down. 3rd and 4th wks Monday: weight-bearing lunge + split-leg SJ + dumbbell bench press + kneeling forward medicine ball; Thursday: military press + push press + reverse grip loaded pull-ups + elastic band pull-ups. RT Program (6 series × reps 6~10): 1st and 2nd wks Monday: squat + barbell BP; Thursday: deadlift + loaded pull-ups. 3rd and 4th wks Monday: weight-bearing lunge + dumbbell BP; Thursday: military press + loaded pull-ups.	CT showed significantly greater improvements in strength and power of firefighters compared to RT, thereby better enhancing their skills for professional activities.

^A^, according to the definition provided by the World Health Organization [25]. Key: %FM, relative fat mass; ♀, female; ♂, male; ♂♀, male and female; ACSM, American College of Sports Medicine; am, ante meridiem; AMRAP, “as many rounds as possible”; BB, barbell; BMI, body mass index; BMT, basic military training; BO, bent over; BP, blood pressure; BW, body mass or body weight; CG, control group; CLA, classical training; CPG, Cyclic-progressive group; CT, complex training; d, day; d/wk, day/week; DB, dumbbells; DXA, dual-energy X-ray absorptiometry EG, experimental; FMS, functional movement screen; ft, foot; GSIP, goal setting and implementation planning; HBD, hex-bar deadlift; HIFT, high-intensity functional training; HR, heart rate; HRR, heart rate reserve; KB, kettlebell; KPI, Key Performance Indicator; lb, libra; LEO, Law enforcement officers; MIC, micro-training; NASM, National Academy of Sport Medicine; NCPG, Non-cyclic progressive group; PAT, physical abilities test; pm, post meridiem; PO, police officer; PPE, personal protective equipment; PPO, peak power output; pQCT, peripheral quantitative computed tomography; PT, periodized training; PTH, Parathyroid hormone; PTM, power training machine; R, rest; RaT, randomized training; reps, repetitions; RER, respiratory exchange ratio; RM, repetition maximum; ROTC, Reserve Officers’ Training Corps; RT, resistance training; s, seconds; SA, single arm; SCBA, self-contained breathing apparatus; SEG, supervised exercise group; SFGT, simulated fire ground test; SJ, squat jump; TV, television; UAE, United Arab Emirates; USA, United States of America; VJ, vertical jump; *V*O_2_, maximum rate of oxygen consumption; wks, weeks; yds, yards; YMCA, YMCA step test (the 3-min step test, also known as the YMCA, Canadian, or Harvard step test).

**Table 4:** Physical training programs distributions.

Study	Circuit-Training	Weight Training	Cardio	Calisthenics	HIFT
Rossomanno et al., 2012 [13]	X	-	X	X	-
Wood and Krüger, 2013 [14]	-	X	X	-	-
Crawley et al., 2015 [3]	X	X	X	X	-
Pawlak et al., 2015 [15]	X	X	X	X	-
Cocke et al., 2016 [16]	-	X	X	X	X
Campos et al., 2017 [17]	X	X	X	-	-
Bycura et al., 2018 [18]	-	-	X	-	-
Čvorović et al., 2018 [19]	X	X	X	X	-
Jafari et al., 2018 [20]	-	X	-	-	-
Kudryavtsev et al., 2018 [21]	-	X	-	-	-
Reau et al., 2018 [21]	X	X	X	-	-
Kilen et al., 2020 [23]	X	-	X	X	-
Lan et al., 2020 [24]	-	X	X	X	-
Lockie et al., 2020 [25]	X	-	X	X	-
Sokoloski et al., 2020 [26]	X	-	X	X	-
Stone et al., 2020 [27]	-	X	X	-	-
Bonder et al., 2021 [28]	-	-	-	-	-
Chizewski et al., 2021 [29]	X	-	X	X	X
Judge et al., 2021 [30]	X	X	-	X	-
Silva et al., 2021 [31]	X	X	X	-	-
Stojković et al., 2021 [32]	X	X	X	X	-
Baker et al., 2022 [33]	X	-	X	X	X
Liu et al., 2022 [34]	-	X	-	X	-
Total	14	15	18	14	3

Key: HIFT, high-intensity functional training.

**Table 5:** Effect size (Cohen’s *d*) and effect size correlation (*r*) of physical training programs on fitness measures.

Study	n	Sex	Duration (wks)	Fitness Test	Pré-		Post-		Pré- vs. Post-	Cohen’s *d*	Effect-Size *r* ^C^
					Mean	SD	Mean	SD			
Rossomanno et al., 2012 [13]	165	Male and Female	25	Physical activity test	-	-	-	-	-	-	-
Wood and Krüguer, 2013 [14]—Non-cyclic progressive group	73	Male	12	2.4-km run (min)	8.60	1.00	9.10	0.80	0.50	−0.55	−0.27
Wood and Krüguer, 2013 [14]—Non-cyclic progressive group	73	Male	12	Push-ups 120 s (reps)	39.20	12.90	53.60	11.30	14.40	−1.19	−0.51
Wood and Krüguer, 2013 [14]—Non-cyclic progressive group	73	Male	12	Sit-ups 120 s (reps)	44.80	2.20	72.40	15.10	27.60	−2.56	−0.79
Wood and Krüguer, 2013 [14]—Non-cyclic progressive group	73	Male	12	Shuttle runs—10 × 22 m (s)	51.20	4.10	48.20	4.20	−3.00	0.72	0.34
Wood and Krüguer, 2013 [14]—Non-cyclic progressive group	115	Female	12	2.4-km run (min)	13.20	2.40	12.60	1.60	−0.60	0.29	0.15
Wood and Krüguer, 2013 [14]—Non-cyclic progressive group	115	Female	12	Push-ups 120 s (reps)	43.10	13.40	58.50	14.00	15.40	−1.12	−0.49
Wood and Krüguer, 2013 [14]—Non-cyclic progressive group	115	Female	12	Sit-ups 120 s (reps)	28.50	14.70	56.40	18.70	27.90	−1.66	−0.64
Wood and Krüguer, 2013 [14]—Non-cyclic progressive group	115	Female	12	Shuttle runs—10 × 22 m (s)	63.10	6.70	60.40	6.40	−2.70	0.41	0.20
Wood and Krüguer, 2013 [14]—Cyclic-progressive group	100	Male	12	2.4-km run (min)	10.50	1.00	9.20	0.60	−1.30	1.58	0.62
Wood and Krüguer, 2013 [14]—Cyclic-progressive group	100	Male	12	Push-ups 120 s (reps)	31.50	9.00	60.10	11.10	28.60	−2.83	−0.82
Wood and Krüguer, 2013 [14]—Cyclic-progressive group	100	Male	12	Sit-ups 120 s (reps)	34.50	10.10	65.40	14.20	30.90	−2.51	−0.78
Wood and Krüguer, 2013 [14]—Cyclic-progressive group	100	Male	12	Shuttle runs—10 × 22 m (s)	55.40	3.60	53.10	3.10	−2.30	0.68	0.32
Wood and Krüguer, 2013 [14]—Cyclic-progressive group	85	Female	12	2.4-km run (min)	16.60	1.80	13.40	1.40	−3.20	1.98	0.70
Wood and Krüguer, 2013 [14]—Cyclic-progressive group	85	Female	12	Push-ups 120 s (reps)	33.00	10.40	56.30	13.70	23.30	−1.92	−0.69
Wood and Krüguer, 2013 [14]—Cyclic-progressive group	85	Female	12	Sit-ups 120 s (reps)	24.40	10.00	49.80	14.30	25.40	−2.06	−0.71
Wood and Krüguer, 2013 [14]—Cyclic-progressive group	85	Female	12	Shuttle runs—10 × 22 m (s)	67.50	8.10	65.10	6.00	−2.40	0.34	0.17
Crawley et al., 2015 [3]	68	Male and Female	16	Wingate PPO (W/kg)	10.10	1.70	10.80	1.60	0.70	−0.42	−0.21
Crawley et al., 2015 [3]	68	Male and Female	16	Sprint (s)	5.61	0.50	5.40	0.30	−0.21	0.51	0.25
Crawley et al., 2015 [3]	68	Male and Female	16	*t*-test (s)	11.50	1.30	11.00	1.10	−0.50	0.42	0.20
Crawley et al., 2015 [3]	68	Male and Female	16	Handgrip—right hand (kg)	53.00	11.00	-	-	-	-	-
Crawley et al., 2015 [3]	68	Male and Female	16	Handgrip—left hand (kg)	50.00	12.00	-	-	-	-	-
Crawley et al., 2015 [3]	68	Male and Female	16	Sit-and-reach (cm)	28.40	8.30	-	-	-	-	-
Crawley et al., 2015 [3]	68	Male and Female	16	Vertical jump (cm)	56.50	10.50	61.20	10.20	4.70	−0.45	−0.22
Crawley et al., 2015 [3]	68	Male and Female	16	Push-ups 60 s (reps)	44.00	14.00	51.00	15.00	7.00	−0.48	−0.23
Crawley et al., 2015 [3]	68	Male and Female	16	Sit-ups 60 s (reps)	42.00	8.00	49.00	7.00	7.00	−0.93	−0.42
Crawley et al., 2015 [3]	68	Male and Female	16	Shuttle run—1/2 mile (s)	233.00	19.00	221.00	17.00	−12.00	0.67	0.32
Crawley et al., 2015 [3]	68	Male and Female	16	Arm crank PPO (W/kg)	2.20	0.70	2.40	0.50	0.20	−0.33	−0.16
Pawlak et al., 2015 [15]—Supervised exercise group	11	Male	12	Handgrip—mean left/right hand (kg)	46.50	11.30	50.00	8.60	3.50	−0.35	−0.17
Pawlak et al., 2015 [15]—Supervised exercise group	11	Male	12	Flexibility (cm)	22.60	11.70	24.70	12.50	2.10	−0.17	−0.09
Pawlak et al., 2015 [15]—Supervised exercise group	11	Male	12	Peak *V*O_2_ (mL/kg/min)	41.50	4.20	43.80	4.80	2.30	−0.51	−0.25
Pawlak et al., 2015 [15]—Supervised exercise group	11	Male	12	Absolute *V*O_2_ (lO_2_/min)	3.83	0.51	3.88	0.50	0.05	−0.10	−0.05
Pawlak et al., 2015 [15]—Control group	9	Male	12	Handgrip—mean left/right hand (kg)	49.30	5.90	52.20	5.40	2.90	−0.51	−0.25
Pawlak et al., 2015 [15]—Control group	9	Male	12	Flexibility (cm)	23.20	7.70	24.50	9.80	1.30	−0.15	−0.07
Pawlak et al., 2015 [15]—Control group	9	Male	12	Peak *V*O_2_ (mL/kg/min)	43.00	4.90	42.40	5.00	−0.60	0.12	0.06
Pawlak et al., 2015 [15]—Control group	9	Male	12	Absolute *V*O_2_ (lO_2_/min)	3.66	0.22	3.63	0.19	−0.03	0.15	0.07
Cocke et al., 2016 [16]—Randomized training group	50	Male and Female	25	Bench press (kg)	88.45	23.69	101.09	21.61	12.64	−0.56	−0.27
Cocke et al., 2016 [16]—Randomized training group	50	Male and Female	25	Push-ups 60 s (reps)	48.96	15.15	70.56	11.99	21.60	−1.58	−0.62
Cocke et al., 2016 [16]—Randomized training group	50	Male and Female	25	Sit-ups 60 s (reps)	33.96	9.02	46.44	5.40	12.48	−1.68	−0.64
Cocke et al., 2016 [16]—Randomized training group	50	Male and Female	25	Vertical jump (cm)	55.32	10.68	62.69	8.64	7.37	−0.76	−0.35
Cocke et al., 2016 [16]—Randomized training group	50	Male and Female	25	Vertical jump—power (W)	5235.01	866.29	5608.97	707.13	373.96	−0.47	−0.23
Cocke et al., 2016 [16]—Randomized training group	50	Male and Female	25	2.4-km run (s)	752.40	84.6	667.20	70.2	−85.20	1.71	0.48
Cocke et al., 2016 [16]—Randomized training group	50	Male and Female	25	300-m run (s)	53.36	4.98	48.23	3.96	−5.13	1.14	0.50
Cocke et al., 2016 [16]—Periodized training group	11	Male and Female	25	Bench press (kg)	106.20	15.15	113.02	20.07	6.82	−0.38	−0.19
Cocke et al., 2016 [16]—Periodized training group	11	Male and Female	25	Push-ups 60 s (reps)	53.45	14.40	70.18	13.67	16.73	−1.19	−0.51
Cocke et al., 2016 [16]—Periodized training group	11	Male and Female	25	Sit-ups 60 s (reps)	42.27	8.51	51.82	5.23	9.55	−1.35	−0.56
Cocke et al., 2016 [16]—Periodized training group	11	Male and Female	25	Vertical jump (cm)	64.54	8.59	64.31	9.22	−0.23	0.03	0.01
Cocke et al., 2016 [16]—Periodized training group	11	Male and Female	25	Vertical jump—Power (W)	5979.54	762.59	5810.48	934.87	−169.06	0.20	0.10
Cocke et al., 2016 [16]—Periodized training group	11	Male and Female	25	2.4-km run (s)	689.40	84.6	656.40	71.4	−33.00	0.42	0.21
Cocke et al., 2016 [16]—Periodized training group	11	Male and Female	25	300-m run (s)	51.75	4.18	49.81	4.02	−1.94	0.47	0.23
Campos et al., 2017 [17]	130	Male	12	Push-ups 60 s (reps)	21.50	9.00	33.70	9.10	12.20	−1.35	−0.56
Campos et al., 2017 [17]	130	Male	12	Sit-ups 60 s (reps)	35.10	8.50	49.80	7.60	14.70	−1.82	−0.67
Campos et al., 2017 [17]	130	Male	12	Cooper—12 min run (m)	2207.00	319.00	2756.00	217.00	549.00	−2.01	−0.71
Campos et al., 2017 [17]	130	Male	12	Absolute *V*O_2max_ (l.min^−1^)	2.50	0.50	3.40	0.50	0.90	−1.80	−0.67
Bycura et al., 2018 [18]—Control group	8	Male	14	*V*O_2_ (mL/kg/min)	25.22	4.19	27.91	4.00	2.69	−0.29	−0.15
Bycura et al., 2018 [18]—Control group	12	Male	14	*V*O_2_ (mL/kg/min)	25.19	2.84	27.20	3.57	2.01	−0.62	−0.30
Čvorović et al., 2018 [19]	325	Male	12	Push-ups 60 s (reps)	22.73	9.39	36.38	8.87	13.65	−1.48	−0.60
Čvorović et al., 2018 [19]	325	Male	12	Sit-ups 60 s (reps)	30.78	7.19	42.35	7.69	11.57	−1.55	−0.61
Čvorović et al., 2018 [19]	325	Male	12	2.4-km run (s)	762.23	113.22	642.07	44.75	−120.16	1.40	0.57
Jafari et al., 2018 [20]—Experimental group	51	unknown	8	FMS ^A^	10.57	3.44	17.82	1.68	7.25	−2.68	−0.80
Jafari et al., 2018 [20]—Control group	45	unknown	8	FMS ^A^	11.80	3.53	12.11	3.61	0.31	−0.09	−0.04
Kudryavtsev et al., 2018 [21]—Control group	14	Male	5	Push-ups 60 s (reps)	25.23	0.39	29.57	1.44	4.34	−4.11	−0.90
Kudryavtsev et al., 2018 [21]—Control group	14	Male	5	Shuttle run—10 × 10 m (s)	32.83	2.51	31.17	2.23	−1.66	0.70	0.33
Kudryavtsev et al., 2018 [21]—Control group	14	Male	5	Harvard step-test (Fitness Index ^B^)	66.34	2.41	68.52	2.06	2.18	−0.97	−0.44
Kudryavtsev et al., 2018 [21]—Control group	14	Male	5	Handgrip (kg)	48.21	2.34	49.17	2.21	0.96	−0.42	−0.21
Kudryavtsev et al., 2018 [21]—Experimental group	14	Male	5	Push-ups 60 s (reps)	25.02	0.37	31.42	1.56	6.40	−5.65	−0.94
Kudryavtsev et al., 2018 [21]—Experimental group	14	Male	5	Shuttle run—10 × 10 m (s)	33.02	2.64	29.14	2.06	−3.88	1.64	0.63
Kudryavtsev et al., 2018 [21]—Experimental group	14	Male	5	Harvard step-test (Fitness Index ^B^)	67.08	2.17	70.45	2.03	3.37	−1.60	−0.63
Kudryavtsev et al., 2018 [21]—Experimental group	14	Male	5	Handgrip (kg)	48.16	2.13	50.44	2.46	2.28	−0.99	−0.44
Reau et al., 2018 [22]	148	Male	16	Pull-ups—max (reps)	10.10	6.50	13.70	6.80	3.60	−0.54	−0.26
Reau et al., 2018 [22]	148	Male	16	Push-ups 60 s (reps)	47.80	16.20	65.70	14.50	17.90	−1.16	−0.50
Reau et al., 2018 [22]	148	Male	16	Bodyweight Squats 60 s (reps)	49.10	9.80	66.70	8.60	17.60	−1.91	−0.69
Reau et al., 2018 [22]	148	Male	16	2.4-km (1.5 miles) run (min:s)	11.59	0.42	11.13	0.32	−0.46	1.23	0.52
Reau et al., 2018 [22]	148	Male	16	Plank (max)	2.06	1.08	2.55	1.21	0.49	−0.43	−0.21
Kilen et al., 2020 [23]—Micro-training group	95	Male and Female	9	Cooper—12-min run (m)	2556.00	324.00	2785.00	269.00	229.00	−0.77	−0.36
Kilen et al., 2020 [23]—Micro-training group	95	Male and Female	9	20-m shuttle run (m)	919.00	417.00	1139.00	417.00	220.00	−0.53	−0.26
Kilen et al., 2020 [23]—Micro-training group	95	Male and Female	9	Lunges (120 s) (reps)	43.30	11.10	51.80	10.70	8.50	−0.78	−0.36
Kilen et al., 2020 [23]—Micro-training group	95	Male and Female	9	Push-ups (120 s) (reps)	29.20	9.80	31.30	7.70	2.10	−0.24	−0.12
Kilen et al., 2020 [23]—Micro-training group	95	Male and Female	9	Sit-ups (120 s) (reps)	60.10	13.40	68.10	13.10	8.00	−0.60	−0.29
Kilen et al., 2020 [23]—Micro-training group	95	Male and Female	9	Back ex TTE (s)	111.70	45.40	133.80	38.40	22.10	−0.53	−0.25
Kilen et al., 2020 [23]—Micro-training group	95	Male and Female	9	Peak *V*O_2_ (mlO_2_/min)	4164.00	484.00	4436.00	526.00	272.00	−0.54	−0.26
Kilen et al., 2020 [23]—Classical-training group	95	Male and Female	9	Cooper—12-min run (m)	2670.00	263.00	2869.00	229.00	199.00	−0.81	−0.37
Kilen et al., 2020 [23]—Classical-training group	95	Male and Female	9	20-m shuttle run (m)	901.00	387.00	1152.00	442.00	251.00	−0.60	−0.29
Kilen et al., 2020 [23]—Classical-training group	95	Male and Female	9	Lunges (120 s) (reps)	43.50	12.90	49.60	12.00	6.10	−0.49	−0.24
Kilen et al., 2020 [23]—Classical-training group	95	Male and Female	9	Push-ups (120 s) (reps)	29.80	9.20	32.00	8.90	2.20	−0.24	−0.12
Kilen et al., 2020 [23]—Classical-training group	95	Male and Female	9	Sit-ups (120 s) (reps)	61.40	13.70	67.20	15.50	5.80	−0.40	−0.19
Kilen et al., 2020 [23]—Classical-training group	95	Male and Female	9	Static back extension (s)	93.00	32.70	134.60	47.10	41.60	−1.03	−0.46
Kilen et al., 2020 [23]—Classical-training group	95	Male and Female	9	Peak *V*O_2_ (mlO_2_/min)	4167.00	697.00	4284.00	510.00	117.00	−0.19	−0.10
Kilen et al., 2020 [23]—Control group	100	Male and Female	9	Cooper—12-min run (m)	2599.00	329.00	2750.00	214.00	151.00	−0.54	−0.26
Kilen et al., 2020 [23]—Control group	100	Male and Female	9	20-m shuttle run (m)	938.00	349.00	1247.00	414.00	309.00	−0.81	−0.37
Kilen et al., 2020 [23]—Control group	100	Male and Female	9	Lunges (120 s) (reps)	45.40	12.50	50.70	10.90	5.30	−0.45	−0.22
Kilen et al., 2020 [23]—Control group	100	Male and Female	9	Push-ups (120 s) (reps)	25.70	9.10	29.60	8.20	3.90	−0.45	−0.22
Kilen et al., 2020 [23]—Control group	100	Male and Female	9	Sit-ups (120 s) (reps)	59.80	14.20	68.40	14.00	8.60	−0.61	−0.29
Kilen et al., 2020 [23]—Control group	100	Male and Female	9	Static back extension (s)	111.20	40.80	147.00	51.80	35.80	−0.77	−0.36
Kilen et al., 2020 [23]—Control group	100	Male and Female	9	Peak *V*O_2_ (mlO_2_/min)	4361.00	648.00	4832.00	628.00	471.00	−0.74	−0.35
Lan et al., 2020 [24]	92	unknown	16	Push-ups 60 s (reps)	34.00	-	52.50	-	18.50	-	-
Lan et al., 2020 [24]	92	unknown	16	Pull-ups—max (reps)	7.00	-	13.00	-	6.00	-	-
Lan et al., 2020 [24]	92	unknown	16	2.4-km run (s)	732.00	-	660.00	-	−72.00	-	-
Lockie et al., 2020 [25]	23	Male	14	Vertical jump (cm)	57.00	-	59.00	-	2.00	-	-
Lockie et al., 2020 [25]	23	Male	14	Push-ups 60 s (reps)	52.00	-	54.00	-	2.00	-	-
Lockie et al., 2020 [25]	23	Male	14	Sit-ups 60 s (reps)	44.00	-	49.00	-	5.00	-	-
Lockie et al., 2020 [25]	23	Male	14	Lower-back and leg strength (kg)	172.00	-	189.00	-	17.00	-	-
Lockie et al., 2020 [25]	23	Male	14	Handgrip—mean left/right hand (kg)	52.00	-	54.00	-	2.00	-	-
Lockie et al., 2020 [25]	23	Male	14	20-m shuttle run (#)	76.00	-	85.00	-	9.00	-	-
Lockie et al., 2020 [25]	3	Female	14	Vertical jump (cm)	42.00	-	45.00	-	3.00	-	-
Lockie et al., 2020 [25]	3	Female	14	Push-ups 60 s (reps)	35.00	-	41.00	-	6.00	-	-
Lockie et al., 2020 [25]	3	Female	14	Sit-ups 60 s (reps)	42.00	-	52.00	-	10.00	-	-
Lockie et al., 2020 [25]	3	Female	14	Lower-back and leg strength (kg)	119.00	-	130.00	-	11.00	-	-
Lockie et al., 2020 [25]	3	Female	14	Handgrip—mean left/right hand (kg)	38.00	-	42.00	-	4.00	-	-
Lockie et al., 2020 [25]	3	Female	14	20-m shuttle run (#)	43.00	-	63.00	-	20.00	-	-
Sokoloski et al., 2020 [26]	34	Male and Female	25	Sit-and-reach (cm)	57.00	14.70	71.70	16.70	14.70	−0.93	−0.42
Sokoloski et al., 2020 [26]	34	Male and Female	25	Push-ups—max (reps)	29.00	15.00	35.00	16.00	6.00	−0.39	−0.19
Sokoloski et al., 2020 [26]	34	Male and Female	25	Sit-ups 60 s (reps)	22.00	22.00	48.00	26.00	26.00	−1.08	−0.48
Stone et al., 2020 [27]	23	Male	11	Hex-bar 1 RM (kg)	139.60	49.20	159.20	21.70	19.60	−0.51	−0.25
Stone et al., 2020 [27]	23	Male	11	20-m shuttle run (#)	41.00	14.20	66.80	16.30	25.80	−1.69	−0.64
Stone et al., 2020 [27]	23	Male	11	Pull-ups—max (reps)	8.83	4.90	11.70	5.10	2.87	−0.57	−0.28
Stone et al., 2020 [27]	23	Male	11	Handgrip—right hand (kg)	55.80	6.80	53.60	7.80	−2.20	0.30	0.15
Stone et al., 2020 [27]	23	Male	11	Handgrip—left hand (kg)	54.30	6.70	52.70	6.90	−1.60	0.24	0.12
Stone et al., 2020 [27]	23	Male	11	Vertical jump (cm)	61.20	8.90	61.50	7.10	0.30	−0.04	−0.02
Bonder et al., 2021 [28]	7	Male	4	HBD 3 RM (p)	336.43	77.98	352.14	74.32	15.71	−0.21	−0.10
Bonder et al., 2021 [28]	7	Male	4	20-m sprint (s)	3.25	0.23	3.21	0.22	−0.04	0.18	0.09
Chizewski et al., 2021 [29]	89	Male	7	2.4-km run (s)	786.00	108	702.00	90.0	−84.00	1.10	0.39
Chizewski et al., 2021 [29]	89	Male	7	Push-ups 60 s (reps)	41.90	12.40	45.30	5.20	3.40	−0.36	−0.18
Chizewski et al., 2021 [29]	89	Male	7	Sit-ups 60 s (reps)	31.40	6.10	38.30	7.80	6.90	−0.99	−0.44
Chizewski et al., 2021 [29]	89	Male	7	Bench press 36-kg—60 s (reps)	30.40	11.60	35.60	11.60	5.20	−0.45	−0.22
Chizewski et al., 2021 [29]	89	Male	7	Sit-and-reach (cm)	7.60	7.20	9.80	7.10	2.20	−0.31	−0.15
Chizewski et al., 2021 [29]	89	Male	7	Vertical jump (in)	24.30	3.70	24.40	4.10	0.10	−0.03	−0.01
Chizewski et al., 2021 [29]	89	Male	7	Kiser sled (s)	44.30	17.30	35.20	8.90	−9.10	0.66	0.31
Chizewski et al., 2021 [29]	89	Male	7	SCBA crawl (s)	44.20	11.70	35.20	8.90	−9.00	0.87	0.40
Chizewski et al., 2021 [29]	89	Male	7	Victim drag (s)	22.50	5.90	19.40	4.60	−3.10	0.59	0.28
Chizewski et al., 2021 [29]	89	Male	7	Hose advance (s)	15.20	3.70	13.90	3.70	−1.30	0.35	0.17
Chizewski et al., 2021 [29]	89	Male	7	Equipment carry (s)	20.90	3.20	19.30	3.10	−1.60	0.51	0.25
Chizewski et al., 2021 [29]	89	Male	7	Ladder raise (s)	7.40	2.20	6.50	1.50	−0.90	0.48	0.23
Chizewski et al., 2021 [29]	89	Male	7	Challenge total (s)	240.20	41.20	192.40	41.60	−47.80	1.15	0.50
Judge et al., 2021 [30]	38	unknown	8	Push-ups 60 s (reps)	43.00	6.14	50.00	6.15	7.00	−1.14	−0.49
Judge et al., 2021 [30]	38	unknown	8	Sit-ups 60 s (reps)	41.00	6.80	48.00	6.70	7.00	−1.04	−0.46
Silva et al., 2021 [31]—Experimental group 1	60	Male	24	Cooper—12-min run (m)	2288.20	247.00	2346.20	252.40	58.00	−0.23	−0.12
Silva et al., 2021 [31]—Experimental group 2	60	Male	24	Cooper—12-min run (m)	2365.40	372.00	2405.70	338.30	40.30	−0.11	−0.06
Silva et al., 2021 [31]—Control group	60	Male	24	Cooper—12-min run (m)	2159.10	218.50	2156.90	215.80	−2.20	0.01	0.01
Stojković et al., 2021 [32]	46	Male	10	Push-ups 60 s (reps)	14.10	7.90	28.70	8.40	14.60	−1.79	−0.67
Stojković et al., 2021 [32]	46	Male	10	Sit-ups 60 s (reps)	23.40	6.50	36.40	5.00	13.00	−2.24	−0.75
Stojković et al., 2021 [32]	46	Male	10	2.4-km run (s)	1027.80	191.80	693.60	86.80	−334.20	2.24	0.75
Stojković et al., 2021 [32]	46	Male	10	*t*-Test (s)	16.22	1.78	13.90	1.50	−2.32	1.41	0.58
Baker et al., 2022 [33]—Control group	18	Male and Female	8	1 RM back squat (kg)	77.90	36.00	80.60	35.00	2.70	−0.08	−0.04
Baker et al., 2022 [33]—Control group	18	Male and Female	8	1 RM leg press (kg)	257.10	106.80	284.90	112.20	27.80	−0.25	−0.13
Baker et al., 2022 [33]—Experimental group	18	Male and Female	8	1 RM back squat (kg)	80.00	30.60	82.80	30.00	2.80	−0.09	−0.05
Baker et al., 2022 [33]—Experimental group	18	Male and Female	8	1 RM leg press (kg)	251.90	80.40	283.70	80.70	31.80	−0.39	−0.19
Liu et al., 2022 [34]—Control group	15	Male	12	100-m load-bearing run (s)	19.24	1.53	17.85	1.05	−1.39	1.06	0.47
Liu et al., 2022 [34]—Control group	15	Male	12	60-m shoulder ladder run (s)	12.71	0.84	11.58	0.84	−1.13	1.35	0.56
Liu et al., 2022 [34]—Control group	15	Male	12	5 × 20-m shuttle run (s)	49.48	2.75	48.92	3.21	−0.56	0.19	0.09
Liu et al., 2022 [34]—Control group	15	Male	12	4th-floor CR (s)	28.51	6.39	24.41	5.82	−4.10	0.67	0.32
Liu et al., 2022 [34]—Control group	15	Male	12	1 RM back squat (kg)	100.67	7.99	110.67	7.99	10.00	−1.25	−0.53
Liu et al., 2022 [34]—Control group	15	Male	12	1 RM bench press (kg)	73.33	9.00	90.00	8.02	16.67	−1.96	−0.70
Liu et al., 2022 [34]—Control group	15	Male	12	Vertical jump (Abalakov) (cm)	37.53	4.31	42.53	5.37	5.00	−1.03	−0.46
Liu et al., 2022 [34]—Control group	15	Male	12	Seated medicine ball throw—3 kg (m)	4.06	0.43	4.80	0.22	0.74	−2.17	−0.73
Liu et al., 2022 [34]—Resistance training group	15	Male	12	100-m load-bearing run (s)	19.25	1.41	18.24	1.30	−1.01	0.74	0.35
Liu et al., 2022 [34]—Resistance training group	15	Male	12	60-m shoulder ladder run (s)	12.84	1.31	12.50	1.33	−0.34	0.26	0.13
Liu et al., 2022 [34]—Resistance training group	15	Male	12	5 × 20 m shuttle run (s)	48.09	5.77	47.30	3.14	−0.79	0.17	0.08
Liu et al., 2022 [34]—Resistance training group	15	Male	12	4th-floor CR (s)	30.40	7.69	27.60	4.88	−2.80	0.43	0.21
Liu et al., 2022 [34]—Resistance training group	15	Male	12	1 RM back squat (kg)	100.33	10.93	109.67	11.87	9.34	−0.82	−0.38
Liu et al., 2022 [34]—Resistance training group	15	Male	12	1 RM bench press (kg)	74.33	12.52	85.67	10.67	11.34	−0.97	−0.44
Liu et al., 2022 [34]—Resistance training group	15	Male	12	Vertical jump (Abalakov) (cm)	37.60	3.09	37.80	3.03	0.20	−0.07	−0.03
Liu et al., 2022 [34]—Resistance training group	15	Male	12	Seated medicine ball throw—3 kg (m)	4.16	0.43	4.33	0.45	0.17	−0.39	−0.19

Key: -, not available; #, number of shuttles completed; 1 RM, one repetition maximum; HBD, hex-bar deadlift; p, pounds; PPO, peak power output; SCBA, self-contained breathing apparatus; SD, standard deviation; wks, weeks. ^A^, functional movement screen components: (A) deep squat, (B) hurdle step, (C) in-line lunge, (D) shoulder mobility, (E) active straight leg. ^B^, *Fitness Index* = (100 × test duration in seconds) divided by (2 × sum of heart beats in the recovery periods). ^C^, effect sizes (d): less than 0.2 was considered a trivial effect; 0.2 to 0.6 a small effect; 0.6 to 1.2 a moderate effect; 1.2 to 2.0 a large effect; 2.0 to 4.0 a very large effect; 4.0 and above an extremely large effect.

## Data Availability

Not applicable.
