# Correction: Rasteiro et al. Physical Training Programs for Tactical Populations: Brief Systematic Review. *Healthcare* 2023, *11*, 967

**DOI:** 10.3390/healthcare11182470

**Published:** 2023-09-05

**Authors:** André Rasteiro, Vanessa Santos, Luís Miguel Massuça

**Affiliations:** 1 Higher Institute of Police Sciences and Internal Security, 1300-663 Lisbon, Portugal; 2 Exercise and Health Laboratory, CIPER, Faculdade de Motricidade Humana, Universidade de Lisboa, 1495-751 Cruz Quebrada, Portugal; 3 KinesioLab, Research Unit in Human Movement Analysis, Instituto Piaget, 2805-059 Almada, Portugal; 4 ICPOL Research Center, Higher Institute of Police Sciences and Internal Security, 1300-663 Lisbon, Portugal; 5 Research Center for Sport, Physical Education, Exercise and Health, CIDEFES, Universidade Lusófona, 1749-024 Lisbon, Portugal; 6 CIFI2D, Faculty of Sport, Universidade do Porto, 4200-450 Porto, Portugal

## Error in Table

In the original publication [1], there was a mistake in Table 5 as published. The results of studies [16,29] in the 2.4 km run test (s) present the SD in minutes (it should be in seconds). This SD was used to calculate the effect sizes, which resulted in inflated values. The corrected Table 5 appears below.

The authors state that the scientific conclusions are unaffected. This correction was approved by the Academic Editor. The original publication has also been updated.

## Figures and Tables

**Table 5:** Effect size (Cohen’s *d*) and effect size correlation (*r*) of physical training programs on fitness measures.

Study	n	Sex	Duration (wks)	Fitness Test	Pré-		Post-		Pré- vs. Post-	Cohen’s *d*	Effect-Size r ^C^
					Mean	SD	Mean	SD			
Rossomanno et al., 2012 [13]	165	Male and Female	25	Physical activity test	-	-	-	-	-	-	-
Wood and Krüguer, 2013 [14]—Non-cyclic progressive group	73	Male	12	2.4-km run (min)	8.60	1.00	9.10	0.80	0.50	−0.55	−0.27
Wood and Krüguer, 2013 [14]—Non-cyclic progressive group	73	Male	12	Push-ups 120 s (reps)	39.20	12.90	53.60	11.30	14.40	−1.19	−0.51
Wood and Krüguer, 2013 [14]—Non-cyclic progressive group	73	Male	12	Sit-ups 120 s (reps)	44.80	2.20	72.40	15.10	27.60	−2.56	−0.79
Wood and Krüguer, 2013 [14]—Non-cyclic progressive group	73	Male	12	Shuttle runs—10 × 22 m (s)	51.20	4.10	48.20	4.20	−3.00	0.72	0.34
Wood and Krüguer, 2013 [14]—Non-cyclic progressive group	115	Female	12	2.4-km run (min)	13.20	2.40	12.60	1.60	−0.60	0.29	0.15
Wood and Krüguer, 2013 [14]—Non-cyclic progressive group	115	Female	12	Push-ups 120 s (reps)	43.10	13.40	58.50	14.00	15.40	−1.12	−0.49
Wood and Krüguer, 2013 [14]—Non-cyclic progressive group	115	Female	12	Sit-ups 120 s (reps)	28.50	14.70	56.40	18.70	27.90	−1.66	−0.64
Wood and Krüguer, 2013 [14]—Non-cyclic progressive group	115	Female	12	Shuttle runs—10 × 22 m (s)	63.10	6.70	60.40	6.40	−2.70	0.41	0.20
Wood and Krüguer, 2013 [14]—Cyclic-progressive group	100	Male	12	2.4-km run (min)	10.50	1.00	9.20	0.60	−1.30	1.58	0.62
Wood and Krüguer, 2013 [14]—Cyclic-progressive group	100	Male	12	Push-ups 120 s (reps)	31.50	9.00	60.10	11.10	28.60	−2.83	−0.82
Wood and Krüguer, 2013 [14]—Cyclic-progressive group	100	Male	12	Sit-ups 120 s (reps)	34.50	10.10	65.40	14.20	30.90	−2.51	−0.78
Wood and Krüguer, 2013 [14]—Cyclic-progressive group	100	Male	12	Shuttle runs—10 × 22 m (s)	55.40	3.60	53.10	3.10	−2.30	0.68	0.32
Wood and Krüguer, 2013 [14]—Cyclic-progressive group	85	Female	12	2.4-km run (min)	16.60	1.80	13.40	1.40	−3.20	1.98	0.70
Wood and Krüguer, 2013 [14]—Cyclic-progressive group	85	Female	12	Push-ups 120 s (reps)	33.00	10.40	56.30	13.70	23.30	−1.92	−0.69
Wood and Krüguer, 2013 [14]—Cyclic-progressive group	85	Female	12	Sit-ups 120 s (reps)	24.40	10.00	49.80	14.30	25.40	−2.06	−0.71
Wood and Krüguer, 2013 [14]—Cyclic-progressive group	85	Female	12	Shuttle runs—10 × 22 m (s)	67.50	8.10	65.10	6.00	−2.40	0.34	0.17
Crawley et al., 2015 [3]	68	Male and Female	16	Wingate PPO (W/kg)	10.10	1.70	10.80	1.60	0.70	−0.42	−0.21
Crawley et al., 2015 [3]	68	Male and Female	16	Sprint (s)	5.61	0.50	5.40	0.30	−0.21	0.51	0.25
Crawley et al., 2015 [3]	68	Male and Female	16	*t*-test (s)	11.50	1.30	11.00	1.10	−0.50	0.42	0.20
Crawley et al., 2015 [3]	68	Male and Female	16	Handgrip—right hand (kg)	53.00	11.00	-	-	-	-	-
Crawley et al., 2015 [3]	68	Male and Female	16	Handgrip—left hand (kg)	50.00	12.00	-	-	-	-	-
Crawley et al., 2015 [3]	68	Male and Female	16	Sit-and-reach (cm)	28.40	8.30	-	-	-	-	-
Crawley et al., 2015 [3]	68	Male and Female	16	Vertical jump (cm)	56.50	10.50	61.20	10.20	4.70	−0.45	−0.22
Crawley et al., 2015 [3]	68	Male and Female	16	Push-ups 60 s (reps)	44.00	14.00	51.00	15.00	7.00	−0.48	−0.23
Crawley et al., 2015 [3]	68	Male and Female	16	Sit-ups 60 s (reps)	42.00	8.00	49.00	7.00	7.00	−0.93	−0.42
Crawley et al., 2015 [3]	68	Male and Female	16	Shuttle run—1/2 mile (s)	233.00	19.00	221.00	17.00	−12.00	0.67	0.32
Crawley et al., 2015 [3]	68	Male and Female	16	Arm crank PPO (W/kg)	2.20	0.70	2.40	0.50	0.20	−0.33	−0.16
Pawlak et al., 2015 [15]—Supervised exercise group	11	Male	12	Handgrip—mean left/right hand (kg)	46.50	11.30	50.00	8.60	3.50	−0.35	−0.17
Pawlak et al., 2015 [15]—Supervised exercise group	11	Male	12	Flexibility (cm)	22.60	11.70	24.70	12.50	2.10	−0.17	−0.09
Pawlak et al., 2015 [15]—Supervised exercise group	11	Male	12	Peak *V*O_2_ (mL/kg/min)	41.50	4.20	43.80	4.80	2.30	−0.51	−0.25
Pawlak et al., 2015 [15]—Supervised exercise group	11	Male	12	Absolute *V*O_2_ (lO_2_/min)	3.83	0.51	3.88	0.50	0.05	−0.10	−0.05
Pawlak et al., 2015 [15]—Control group	9	Male	12	Handgrip—mean left/right hand (kg)	49.30	5.90	52.20	5.40	2.90	−0.51	−0.25
Pawlak et al., 2015 [15]—Control group	9	Male	12	Flexibility (cm)	23.20	7.70	24.50	9.80	1.30	−0.15	−0.07
Pawlak et al., 2015 [15]—Control group	9	Male	12	Peak *V*O_2_ (mL/kg/min)	43.00	4.90	42.40	5.00	−0.60	0.12	0.06
Pawlak et al., 2015 [15]—Control group	9	Male	12	Absolute *V*O_2_ (lO_2_/min)	3.66	0.22	3.63	0.19	−0.03	0.15	0.07
Cocke et al., 2016 [16]—Randomized training group	50	Male and Female	25	Bench press (kg)	88.45	23.69	101.09	21.61	12.64	−0.56	−0.27
Cocke et al., 2016 [16]—Randomized training group	50	Male and Female	25	Push-ups 60 s (reps)	48.96	15.15	70.56	11.99	21.60	−1.58	−0.62
Cocke et al., 2016 [16]—Randomized training group	50	Male and Female	25	Sit-ups 60 s (reps)	33.96	9.02	46.44	5.40	12.48	−1.68	−0.64
Cocke et al., 2016 [16]—Randomized training group	50	Male and Female	25	Vertical jump (cm)	55.32	10.68	62.69	8.64	7.37	−0.76	−0.35
Cocke et al., 2016 [16]—Randomized training group	50	Male and Female	25	Vertical jump—power (W)	5235.01	866.29	5608.97	707.13	373.96	−0.47	−0.23
Cocke et al., 2016 [16]—Randomized training group	50	Male and Female	25	2.4-km run (s)	752.40	84.6	667.20	70.2	−85.20	1.71	0.48
Cocke et al., 2016 [16]—Randomized training group	50	Male and Female	25	300-m run (s)	53.36	4.98	48.23	3.96	−5.13	1.14	0.50
Cocke et al., 2016 [16]—Periodized training group	11	Male and Female	25	Bench press (kg)	106.20	15.15	113.02	20.07	6.82	−0.38	−0.19
Cocke et al., 2016 [16]—Periodized training group	11	Male and Female	25	Push-ups 60 s (reps)	53.45	14.40	70.18	13.67	16.73	−1.19	−0.51
Cocke et al., 2016 [16]—Periodized training group	11	Male and Female	25	Sit-ups 60 s (reps)	42.27	8.51	51.82	5.23	9.55	−1.35	−0.56
Cocke et al., 2016 [16]—Periodized training group	11	Male and Female	25	Vertical jump (cm)	64.54	8.59	64.31	9.22	−0.23	0.03	0.01
Cocke et al., 2016 [16]—Periodized training group	11	Male and Female	25	Vertical jump—Power (W)	5979.54	762.59	5810.48	934.87	−169.06	0.20	0.10
Cocke et al., 2016 [16]—Periodized training group	11	Male and Female	25	2.4-km run (s)	689.40	84.6	656.40	71.4	−33.00	0.42	0.21
Cocke et al., 2016 [16]—Periodized training group	11	Male and Female	25	300-m run (s)	51.75	4.18	49.81	4.02	−1.94	0.47	0.23
Campos et al., 2017 [17]	130	Male	12	Push-ups 60 s (reps)	21.50	9.00	33.70	9.10	12.20	−1.35	−0.56
Campos et al., 2017 [17]	130	Male	12	Sit-ups 60 s (reps)	35.10	8.50	49.80	7.60	14.70	−1.82	−0.67
Campos et al., 2017 [17]	130	Male	12	Cooper—12 min run (m)	2207.00	319.00	2756.00	217.00	549.00	−2.01	−0.71
Campos et al., 2017 [17]	130	Male	12	Absolute *V*O_2max_ (l.min^−1^)	2.50	0.50	3.40	0.50	0.90	−1.80	−0.67
Bycura et al., 2018 [18]—Control group	8	Male	14	*V*O_2_ (mL/kg/min)	25.22	4.19	27.91	4.00	2.69	−0.29	−0.15
Bycura et al., 2018 [18]—Control group	12	Male	14	*V*O_2_ (mL/kg/min)	25.19	2.84	27.20	3.57	2.01	−0.62	−0.30
Čvorović et al., 2018 [19]	325	Male	12	Push-ups 60 s (reps)	22.73	9.39	36.38	8.87	13.65	−1.48	−0.60
Čvorović et al., 2018 [19]	325	Male	12	Sit-ups 60 s (reps)	30.78	7.19	42.35	7.69	11.57	−1.55	−0.61
Čvorović et al., 2018 [19]	325	Male	12	2.4-km run (s)	762.23	113.22	642.07	44.75	−120.16	1.40	0.57
Jafari et al., 2018 [20]—Experimental group	51	unknown	8	FMS ^A^	10.57	3.44	17.82	1.68	7.25	−2.68	−0.80
Jafari et al., 2018 [20]—Control group	45	unknown	8	FMS ^A^	11.80	3.53	12.11	3.61	0.31	−0.09	−0.04
Kudryavtsev et al., 2018 [21]—Control group	14	Male	5	Push-ups 60 s (reps)	25.23	0.39	29.57	1.44	4.34	−4.11	−0.90
Kudryavtsev et al., 2018 [21]—Control group	14	Male	5	Shuttle run—10 × 10 m (s)	32.83	2.51	31.17	2.23	−1.66	0.70	0.33
Kudryavtsev et al., 2018 [21]—Control group	14	Male	5	Harvard step-test (Fitness Index ^B^)	66.34	2.41	68.52	2.06	2.18	−0.97	−0.44
Kudryavtsev et al., 2018 [21]—Control group	14	Male	5	Handgrip (kg)	48.21	2.34	49.17	2.21	0.96	−0.42	−0.21
Kudryavtsev et al., 2018 [21]—Experimental group	14	Male	5	Push-ups 60 s (reps)	25.02	0.37	31.42	1.56	6.40	−5.65	−0.94
Kudryavtsev et al., 2018 [21]—Experimental group	14	Male	5	Shuttle run—10 × 10 m (s)	33.02	2.64	29.14	2.06	−3.88	1.64	0.63
Kudryavtsev et al., 2018 [21]—Experimental group	14	Male	5	Harvard step-test (Fitness Index ^B^)	67.08	2.17	70.45	2.03	3.37	−1.60	−0.63
Kudryavtsev et al., 2018 [21]—Experimental group	14	Male	5	Handgrip (kg)	48.16	2.13	50.44	2.46	2.28	−0.99	−0.44
Reau et al., 2018 [22]	148	Male	16	Pull-ups—max (reps)	10.10	6.50	13.70	6.80	3.60	−0.54	−0.26
Reau et al., 2018 [22]	148	Male	16	Push-ups 60 s (reps)	47.80	16.20	65.70	14.50	17.90	−1.16	−0.50
Reau et al., 2018 [22]	148	Male	16	Bodyweight Squats 60 s (reps)	49.10	9.80	66.70	8.60	17.60	−1.91	−0.69
Reau et al., 2018 [22]	148	Male	16	2.4-km (1.5 miles) run (min:s)	11.59	0.42	11.13	0.32	−0.46	1.23	0.52
Reau et al., 2018 [22]	148	Male	16	Plank (max)	2.06	1.08	2.55	1.21	0.49	−0.43	−0.21
Kilen et al., 2020 [23]—Micro-training group	95	Male and Female	9	Cooper—12-min run (m)	2556.00	324.00	2785.00	269.00	229.00	−0.77	−0.36
Kilen et al., 2020 [23]—Micro-training group	95	Male and Female	9	20-m shuttle run (m)	919.00	417.00	1139.00	417.00	220.00	−0.53	−0.26
Kilen et al., 2020 [23]—Micro-training group	95	Male and Female	9	Lunges (120 s) (reps)	43.30	11.10	51.80	10.70	8.50	−0.78	−0.36
Kilen et al., 2020 [23]—Micro-training group	95	Male and Female	9	Push-ups (120 s) (reps)	29.20	9.80	31.30	7.70	2.10	−0.24	−0.12
Kilen et al., 2020 [23]—Micro-training group	95	Male and Female	9	Sit-ups (120 s) (reps)	60.10	13.40	68.10	13.10	8.00	−0.60	−0.29
Kilen et al., 2020 [23]—Micro-training group	95	Male and Female	9	Back ex TTE (s)	111.70	45.40	133.80	38.40	22.10	−0.53	−0.25
Kilen et al., 2020 [23]—Micro-training group	95	Male and Female	9	Peak *V*O_2_ (mlO_2_/min)	4164.00	484.00	4436.00	526.00	272.00	−0.54	−0.26
Kilen et al., 2020 [23]—Classical-training group	95	Male and Female	9	Cooper—12-min run (m)	2670.00	263.00	2869.00	229.00	199.00	−0.81	−0.37
Kilen et al., 2020 [23]—Classical-training group	95	Male and Female	9	20-m shuttle run (m)	901.00	387.00	1152.00	442.00	251.00	−0.60	−0.29
Kilen et al., 2020 [23]—Classical-training group	95	Male and Female	9	Lunges (120 s) (reps)	43.50	12.90	49.60	12.00	6.10	−0.49	−0.24
Kilen et al., 2020 [23]—Classical-training group	95	Male and Female	9	Push-ups (120 s) (reps)	29.80	9.20	32.00	8.90	2.20	−0.24	−0.12
Kilen et al., 2020 [23]—Classical-training group	95	Male and Female	9	Sit-ups (120 s) (reps)	61.40	13.70	67.20	15.50	5.80	−0.40	−0.19
Kilen et al., 2020 [23]—Classical-training group	95	Male and Female	9	Static back extension (s)	93.00	32.70	134.60	47.10	41.60	−1.03	−0.46
Kilen et al., 2020 [23]—Classical-training group	95	Male and Female	9	Peak *V*O_2_ (mlO_2_/min)	4167.00	697.00	4284.00	510.00	117.00	−0.19	−0.10
Kilen et al., 2020 [23]—Control group	100	Male and Female	9	Cooper—12-min run (m)	2599.00	329.00	2750.00	214.00	151.00	−0.54	−0.26
Kilen et al., 2020 [23]—Control group	100	Male and Female	9	20-m shuttle run (m)	938.00	349.00	1247.00	414.00	309.00	−0.81	−0.37
Kilen et al., 2020 [23]—Control group	100	Male and Female	9	Lunges (120 s) (reps)	45.40	12.50	50.70	10.90	5.30	−0.45	−0.22
Kilen et al., 2020 [23]—Control group	100	Male and Female	9	Push-ups (120 s) (reps)	25.70	9.10	29.60	8.20	3.90	−0.45	−0.22
Kilen et al., 2020 [23]—Control group	100	Male and Female	9	Sit-ups (120 s) (reps)	59.80	14.20	68.40	14.00	8.60	−0.61	−0.29
Kilen et al., 2020 [23]—Control group	100	Male and Female	9	Static back extension (s)	111.20	40.80	147.00	51.80	35.80	−0.77	−0.36
Kilen et al., 2020 [23]—Control group	100	Male and Female	9	Peak *V*O_2_ (mlO_2_/min)	4361.00	648.00	4832.00	628.00	471.00	−0.74	−0.35
Lan et al., 2020 [24]	92	unknown	16	Push-ups 60 s (reps)	34.00	-	52.50	-	18.50	-	-
Lan et al., 2020 [24]	92	unknown	16	Pull-ups—max (reps)	7.00	-	13.00	-	6.00	-	-
Lan et al., 2020 [24]	92	unknown	16	2.4-km run (s)	732.00	-	660.00	-	−72.00	-	-
Lockie et al., 2020 [25]	23	Male	14	Vertical jump (cm)	57.00	-	59.00	-	2.00	-	-
Lockie et al., 2020 [25]	23	Male	14	Push-ups 60 s (reps)	52.00	-	54.00	-	2.00	-	-
Lockie et al., 2020 [25]	23	Male	14	Sit-ups 60 s (reps)	44.00	-	49.00	-	5.00	-	-
Lockie et al., 2020 [25]	23	Male	14	Lower-back and leg strength (kg)	172.00	-	189.00	-	17.00	-	-
Lockie et al., 2020 [25]	23	Male	14	Handgrip—mean left/right hand (kg)	52.00	-	54.00	-	2.00	-	-
Lockie et al., 2020 [25]	23	Male	14	20-m shuttle run (#)	76.00	-	85.00	-	9.00	-	-
Lockie et al., 2020 [25]	3	Female	14	Vertical jump (cm)	42.00	-	45.00	-	3.00	-	-
Lockie et al., 2020 [25]	3	Female	14	Push-ups 60 s (reps)	35.00	-	41.00	-	6.00	-	-
Lockie et al., 2020 [25]	3	Female	14	Sit-ups 60 s (reps)	42.00	-	52.00	-	10.00	-	-
Lockie et al., 2020 [25]	3	Female	14	Lower-back and leg strength (kg)	119.00	-	130.00	-	11.00	-	-
Lockie et al., 2020 [25]	3	Female	14	Handgrip—mean left/right hand (kg)	38.00	-	42.00	-	4.00	-	-
Lockie et al., 2020 [25]	3	Female	14	20-m shuttle run (#)	43.00	-	63.00	-	20.00	-	-
Sokoloski et al., 2020 [26]	34	Male and Female	25	Sit-and-reach (cm)	57.00	14.70	71.70	16.70	14.70	−0.93	−0.42
Sokoloski et al., 2020 [26]	34	Male and Female	25	Push-ups—max (reps)	29.00	15.00	35.00	16.00	6.00	−0.39	−0.19
Sokoloski et al., 2020 [26]	34	Male and Female	25	Sit-ups 60 s (reps)	22.00	22.00	48.00	26.00	26.00	−1.08	−0.48
Stone et al., 2020 [27]	23	Male	11	Hex-bar 1 RM (kg)	139.60	49.20	159.20	21.70	19.60	−0.51	−0.25
Stone et al., 2020 [27]	23	Male	11	20-m shuttle run (#)	41.00	14.20	66.80	16.30	25.80	−1.69	−0.64
Stone et al., 2020 [27]	23	Male	11	Pull-ups—max (reps)	8.83	4.90	11.70	5.10	2.87	−0.57	−0.28
Stone et al., 2020 [27]	23	Male	11	Handgrip—right hand (kg)	55.80	6.80	53.60	7.80	−2.20	0.30	0.15
Stone et al., 2020 [27]	23	Male	11	Handgrip—left hand (kg)	54.30	6.70	52.70	6.90	−1.60	0.24	0.12
Stone et al., 2020 [27]	23	Male	11	Vertical jump (cm)	61.20	8.90	61.50	7.10	0.30	−0.04	−0.02
Bonder et al., 2021 [28]	7	Male	4	HBD 3 RM (p)	336.43	77.98	352.14	74.32	15.71	−0.21	−0.10
Bonder et al., 2021 [28]	7	Male	4	20-m sprint (s)	3.25	0.23	3.21	0.22	−0.04	0.18	0.09
Chizewski et al., 2021 [29]	89	Male	7	2.4-km run (s)	786.00	108	702.00	90.0	−84.00	1.10	0.39
Chizewski et al., 2021 [29]	89	Male	7	Push-ups 60 s (reps)	41.90	12.40	45.30	5.20	3.40	−0.36	−0.18
Chizewski et al., 2021 [29]	89	Male	7	Sit-ups 60 s (reps)	31.40	6.10	38.30	7.80	6.90	−0.99	−0.44
Chizewski et al., 2021 [29]	89	Male	7	Bench press 36-kg—60 s (reps)	30.40	11.60	35.60	11.60	5.20	−0.45	−0.22
Chizewski et al., 2021 [29]	89	Male	7	Sit-and-reach (cm)	7.60	7.20	9.80	7.10	2.20	−0.31	−0.15
Chizewski et al., 2021 [29]	89	Male	7	Vertical jump (in)	24.30	3.70	24.40	4.10	0.10	−0.03	−0.01
Chizewski et al., 2021 [29]	89	Male	7	Kiser sled (s)	44.30	17.30	35.20	8.90	−9.10	0.66	0.31
Chizewski et al., 2021 [29]	89	Male	7	SCBA crawl (s)	44.20	11.70	35.20	8.90	−9.00	0.87	0.40
Chizewski et al., 2021 [29]	89	Male	7	Victim drag (s)	22.50	5.90	19.40	4.60	−3.10	0.59	0.28
Chizewski et al., 2021 [29]	89	Male	7	Hose advance (s)	15.20	3.70	13.90	3.70	−1.30	0.35	0.17
Chizewski et al., 2021 [29]	89	Male	7	Equipment carry (s)	20.90	3.20	19.30	3.10	−1.60	0.51	0.25
Chizewski et al., 2021 [29]	89	Male	7	Ladder raise (s)	7.40	2.20	6.50	1.50	−0.90	0.48	0.23
Chizewski et al., 2021 [29]	89	Male	7	Challenge total (s)	240.20	41.20	192.40	41.60	−47.80	1.15	0.50
Judge et al., 2021 [30]	38	unknown	8	Push-ups 60 s (reps)	43.00	6.14	50.00	6.15	7.00	−1.14	−0.49
Judge et al., 2021 [30]	38	unknown	8	Sit-ups 60 s (reps)	41.00	6.80	48.00	6.70	7.00	−1.04	−0.46
Silva et al., 2021 [31]—Experimental group 1	60	Male	24	Cooper—12-min run (m)	2288.20	247.00	2346.20	252.40	58.00	−0.23	−0.12
Silva et al., 2021 [31]—Experimental group 2	60	Male	24	Cooper—12-min run (m)	2365.40	372.00	2405.70	338.30	40.30	−0.11	−0.06
Silva et al., 2021 [31]—Control group	60	Male	24	Cooper—12-min run (m)	2159.10	218.50	2156.90	215.80	−2.20	0.01	0.01
Stojković et al., 2021 [32]	46	Male	10	Push-ups 60 s (reps)	14.10	7.90	28.70	8.40	14.60	−1.79	−0.67
Stojković et al., 2021 [32]	46	Male	10	Sit-ups 60 s (reps)	23.40	6.50	36.40	5.00	13.00	−2.24	−0.75
Stojković et al., 2021 [32]	46	Male	10	2.4-km run (s)	1027.80	191.80	693.60	86.80	−334.20	2.24	0.75
Stojković et al., 2021 [32]	46	Male	10	*t*-Test (s)	16.22	1.78	13.90	1.50	−2.32	1.41	0.58
Baker et al., 2022 [33]—Control group	18	Male and Female	8	1 RM back squat (kg)	77.90	36.00	80.60	35.00	2.70	−0.08	−0.04
Baker et al., 2022 [33]—Control group	18	Male and Female	8	1 RM leg press (kg)	257.10	106.80	284.90	112.20	27.80	−0.25	−0.13
Baker et al., 2022 [33]—Experimental group	18	Male and Female	8	1 RM back squat (kg)	80.00	30.60	82.80	30.00	2.80	−0.09	−0.05
Baker et al., 2022 [33]—Experimental group	18	Male and Female	8	1 RM leg press (kg)	251.90	80.40	283.70	80.70	31.80	−0.39	−0.19
Liu et al., 2022 [34]—Control group	15	Male	12	100-m load-bearing run (s)	19.24	1.53	17.85	1.05	−1.39	1.06	0.47
Liu et al., 2022 [34]—Control group	15	Male	12	60-m shoulder ladder run (s)	12.71	0.84	11.58	0.84	−1.13	1.35	0.56
Liu et al., 2022 [34]—Control group	15	Male	12	5 × 20-m shuttle run (s)	49.48	2.75	48.92	3.21	−0.56	0.19	0.09
Liu et al., 2022 [34]—Control group	15	Male	12	4th-floor CR (s)	28.51	6.39	24.41	5.82	−4.10	0.67	0.32
Liu et al., 2022 [34]—Control group	15	Male	12	1 RM back squat (kg)	100.67	7.99	110.67	7.99	10.00	−1.25	−0.53
Liu et al., 2022 [34]—Control group	15	Male	12	1 RM bench press (kg)	73.33	9.00	90.00	8.02	16.67	−1.96	−0.70
Liu et al., 2022 [34]—Control group	15	Male	12	Vertical jump (Abalakov) (cm)	37.53	4.31	42.53	5.37	5.00	−1.03	−0.46
Liu et al., 2022 [34]—Control group	15	Male	12	Seated medicine ball throw—3 kg (m)	4.06	0.43	4.80	0.22	0.74	−2.17	−0.73
Liu et al., 2022 [34]—Resistance training group	15	Male	12	100-m load-bearing run (s)	19.25	1.41	18.24	1.30	−1.01	0.74	0.35
Liu et al., 2022 [34]—Resistance training group	15	Male	12	60-m shoulder ladder run (s)	12.84	1.31	12.50	1.33	−0.34	0.26	0.13
Liu et al., 2022 [34]—Resistance training group	15	Male	12	5 × 20 m shuttle run (s)	48.09	5.77	47.30	3.14	−0.79	0.17	0.08
Liu et al., 2022 [34]—Resistance training group	15	Male	12	4th-floor CR (s)	30.40	7.69	27.60	4.88	−2.80	0.43	0.21
Liu et al., 2022 [34]—Resistance training group	15	Male	12	1 RM back squat (kg)	100.33	10.93	109.67	11.87	9.34	−0.82	−0.38
Liu et al., 2022 [34]—Resistance training group	15	Male	12	1 RM bench press (kg)	74.33	12.52	85.67	10.67	11.34	−0.97	−0.44
Liu et al., 2022 [34]—Resistance training group	15	Male	12	Vertical jump (Abalakov) (cm)	37.60	3.09	37.80	3.03	0.20	−0.07	−0.03
Liu et al., 2022 [34]—Resistance training group	15	Male	12	Seated medicine ball throw—3 kg (m)	4.16	0.43	4.33	0.45	0.17	−0.39	−0.19

Key: -, not available; #, number of shuttles completed; 1 RM, one repetition maximum; HBD, hex-bar deadlift; p, pounds; PPO, peak power output; SCBA, self-contained breathing apparatus; SD, standard deviation; wks, weeks. ^A^, functional movement screen components: (A) deep squat, (B) hurdle step, (C) in-line lunge, (D) shoulder mobility, (E) active straight leg. ^B^, *Fitness Index* = (100 × test duration in seconds) divided by (2 × sum of heart beats in the recovery periods). ^C^, effect sizes (d): less than 0.2 was considered a trivial effect; 0.2 to 0.6 a small effect; 0.6 to 1.2 a moderate effect; 1.2 to 2.0 a large effect; 2.0 to 4.0 a very large effect; 4.0 and above an extremely large effect.
